# Optimized Distributed Generalized Reed-Solomon Coding with Space-Time Block Coded Spatial Modulation

**DOI:** 10.3390/s22166305

**Published:** 2022-08-22

**Authors:** Chunli Zhao, Fengfan Yang, Daniel Kariuki Waweru, Chen Chen, Hongjun Xu

**Affiliations:** 1 College of Electronic and Information Engineering, Nanjing University of Aeronautics and Astronautics, Nanjing 210016, China; 2 School of Engineering, University of KwaZulu-Natal, King George V Avenue, Durban 4041, South Africa

**Keywords:** generalized Reed–Solomon (GRS), coded cooperation, space-time block coded spatial modulation (STBC-SM)

## Abstract

We present a well-known generalized Reed–Solomon (GRS) code incorporated with space-time block coded spatial modulation (STBC-SM) for wireless networks, which is capable of enjoying coded cooperation between the source and the relay. In the proposed distributed GRS-coded STBC-SM (DGRSC-STBC-SM) scheme, the source and relay nodes use distinct GRS codes. At the relay, we employ the concept of information selection to choose the message symbols from the source for further encoding. Thus, the codewords jointly constructed by the source and relay are generated at the destination. For achieving the best codeword set at the destination, we propose an optimal algorithm at the relay to select partial symbols from the source. To reduce the computational complexity, we propose a more practical algorithm with low complexity. Monte Carlo simulation results show that the proposed scheme using the low-complexity algorithm can achieve near-optimal error performance. Furthermore, our proposed scheme provides better error performance than its corresponding coded non-cooperative counterpart and the existing Reed–Solomon coded cooperative SM (RSCC-SM) scheme under identical conditions.

## 1. Introduction

Multiple-input multiple-output (MIMO) techniques have the potential to combat channel fading [1,2]. One famous MIMO technology is Vertical-Bell Lab Layered Space-Time (V-BLAST) [3]. Unfortunately, due to simultaneous transmission from multiple antennas at the same frequency in V-BLAST, high inter-channel interference (ICI) occurs in the destination. One approach to solving the problem is to utilize spatial modulation (SM) [4]. In SM, the ICI is completely avoided since at each time instant only a single active transmitting antenna is utilized to send the modulated symbol. However, SM cannot exploit the transmit diversity. A novel scheme, termed as space-time block coded SM (STBC-SM) [5], integrates the ideas of space-time block codes (STBCs) and SM. By this means, STBC-SM averts the shortcomings of STBCs and SM while retaining the merits of both schemes. Thus, it is capable of removing ICI and taking advantage of transmit diversity.

Coded cooperation is also an efficient technique that combats channel impairments. The concept of coded cooperation is developed as an evolution of cooperative communications [6] and is an amalgamation of channel coding and cooperative schemes such as amplify-and-forward (AF) [7], decode-and-forward (DF) [8] and compress-and-forward (CF) [9]. A typical coded cooperative scheme is comprised of the source, relay and destination nodes, where the relay overhears the signal of the source node and relays the information to the destination. By the mutual cooperation between the source and relay nodes, a distributed channel code is then constructed at the destination, which delivers better decoding capability than the non-cooperative system. Various distributed binary channel codes, such as distributed turbo code [7], distributed polar code [8] and distributed low-density parity-check (LDPC) code [10] have been investigated to provide cooperation.

However, distributed non-binary channel coding has not been widely studied. In recent years, maximum-distance separable (MDS) codes have turned into an interesting topical subject because they can reach the singleton bound and correct the maximum number of errors [11]. As a special MDS code, the non-binary generalized Reed–Solomon (GRS) code [12] can correct the burst errors. Moreover, the GRS code has a good algebraic structure and wide applications and is drawing attention from many scholars. Most of the previous works only focus on the construction of excellent channel codes with the help of the GRS code. For example, in [13], the authors employed GRS codes to construct quasi-cyclic LDPC (QC-LDPC) codes with girth greater than six, and the experimental results show that the constructed QC-LDPC codes exhibit superior error performance. In [14], several new types of q-ary MDS self-dual codes via GRS codes were constructed with an odd *q*. The literature [15] presented a novel linear complementary-dual MDS code construction through the GRS code and extended the previously known results as well. However, to the best of our knowledge, the achievements about the distributed non-binary GRS coding scheme in cooperative communication scenarios have not been reported. Thus, it is very important and meaningful to investigate the distributed non-binary GRS coding scheme that makes full use of the advantages of coded cooperation and non-binary GRS codes. Moreover, the authors in [16,17,18] have presented that applying the MIMO technology to the distributed channel coding can effectively enhance the error performance of the distributed channel coding scheme. Therefore, the incorporation of the distributed GRS coding and MIMO technology is studied in this paper. As a recently developed MIMO technique, STBC-SM has many advantages over the traditional MIMO schemes. For example, it completely eliminates the ICI and can take advantage of spatial diversity. Thus, this motivates us to explore the distributed GRS-coded STBC-SM (DGRSC-STBC-SM) scheme that combines distributed GRS codes and STBC-SM. In any distributed coding MIMO system, an appropriate encoding strategy at the relay is vital in generating an optimized code in the destination. This brings motivation to proposing efficient symbol-selection algorithms at the relay of the DGRSC-STBC-SM scheme to properly select the partial symbols from the decoded source information symbols.

In this manuscript, the authors propose the DGRSC-STBC-SM scheme. In the proposed DGRSC-STBC-SM scheme, the source and relay employ distinct GRS codes. Compared with the GRS code in the source, the GRS code at the relay has a larger minimum distance. Furthermore, our proposed scheme adopts the principle of symbol selection in the relay to choose partial symbols from the decoded source information symbols. By the cooperation between the source and relay, the destination will generate a joint codeword subset for each selection. In order to obtain the optimized codeword subset with the codewords of a larger minimum weight, two efficient symbol selection algorithms, i.e., the optimal and low-complexity symbol selection algorithms, are proposed at the relay to determine the selection pattern.

Our main contributions are summarized as follows:

- The DGRSC-STBC-SM scheme is first proposed, where the source and relay nodes use different GRS codes. In the DGRSC-STBC-SM scheme, the relay selects partial symbols from the decoded source information symbols for further encoding. For each selection at the relay, the destination then generates a codeword set through the mutual cooperation between the source and relay.
- To construct an optimal codeword set at the destination with the best weight distribution, we propose an optimal symbol selection algorithm at the relay to determine the best selection pattern by which partial symbols are chosen from the decoded source information symbols for further encoding.
- However, for a longer block length code, the complexity of the algorithm is very high. Thus, it is not realistic from a practical perspective. To reduce the computational complexity of the optimal symbol selection algorithm, the low-complexity symbol selection algorithm is then proposed. In the low-complexity symbol selection algorithm, partial source information symbol sequences are considered to determine the optimized selection pattern from the local selection patterns at the relay.

The rest of this paper is organized as follows: Section 2 discusses the works related to our proposed scheme. Section 3 introduces the generalized distributed linear block coded STBC-SM by subset method. The system model of DGRSC- STBC-SM, and two efficient information symbol selection algorithms, are presented in Section 4 and Section 5, respectively. The designed decoding algorithm at the destination is shown in Section 6. Section 7 discusses the simulation results of the investigated schemes. Finally, the conclusions are included in Section 8.

## 2. Related Work

In [19], the RS-coded cooperative system using the adaptive cooperation level was proposed. The authors in [20] presented a distributed RS coding scheme that divides the data into two parts by the use of arithmetic operations. In [21], the authors studied the error performance of the concatenation of RS codes and convolutional codes in cooperative scenarios. In these papers, the designed systems are capable of enjoying the advantages of coded cooperation, but they cannot obtain more spatial diversity. After that, Zhao et al. [16] proposed the RS-coded cooperative SM (RSCC-SM) scheme as the combination of the RS-coded cooperation and the traditional MIMO technology (i.e., SM). Compared to the traditional MIMO techniques, a recently developed STBC-SM [5] has many advantages. For example, it can eliminate the ICI and take advantage of transmit diversity. Based on this reason, Zhao et al. [22] adopted the novel STBC-SM technique [5] and further proposed another RS-coded cooperative STBC-SM scheme (RSCC-STBC-SM) scheme.

As an extension of RS codes, GRS codes have many advantages such as flexible codeword length and parameter vectors. In [12,23], the basic concept of GRS codes was introduced. In [13], Sun et al. used GRS codes to build QC-LDPC codes with a larger girth. In [14], Jin et al. constructed several MDS self-dual codes based on GRS codes. In [15], Chen et al. further extended the work of [14] and constructed a new MDS code. However, in these cases, performance gains generated by the coded cooperation and MIMO technique cannot be obtained. Inspired by the analysis, we propose the DGRSC-STBC-SM scheme. In recent [24], Guo et al. introduced that adopting the appropriate encoding method at the relay helps the destination to generate an optimized codeword set. This motivates us to design an optimized DGRSC-STBC-SM scheme by employing the proper encoding strategy at the relay.

In our proposed DGRSC-STBC-SM scheme, two efficient symbol selection algorithms are presented at the relay to properly select the symbols from the decoded source information symbols for further encoding, such that the destination generates an optimized codeword set with a better weight distribution. Different from the state of the art, the optimized DGRSC-STBC-SM scheme achieves some advancements: (1) The GRS code is first applied in the cooperative systems. (2) The DGRSC-STBC-SM scheme combines the bene-fits of the novel STBC-SM and distributed GRS coding. (3) In the DGRSC-STBC-SM scheme, an optimized codeword set is generated at the destination. Thus, the optimized DGRSC-STBC-SM scheme can achieve performance advantages as compared to the state of the art.

## 3. Generalized Distributed Channel Coding Combined with STBC-SM Based on Subset Method

This section introduces the general design of distributed channel coding combined with STBC-SM based on the subset method. The details are discussed as follows.

### 3.1. Distributed Channel Coding Based on the Subset Method

**Figure 1:**
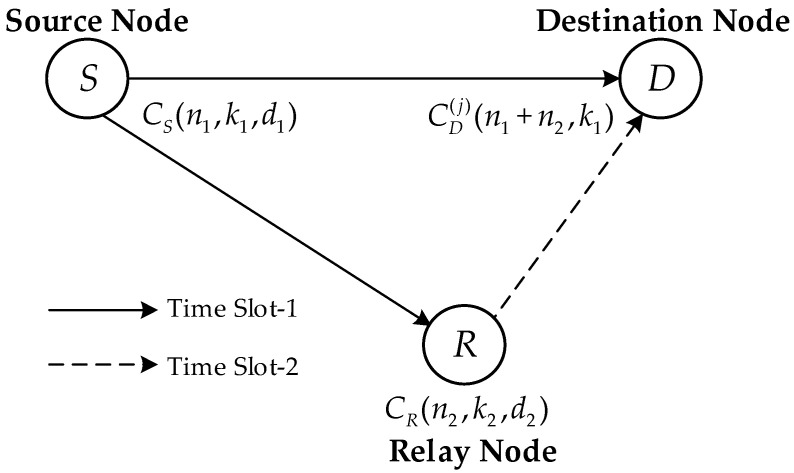
Generalized distributed linear block coding scheme.

Figure 1 shows a typical coded cooperative scheme with three terminals, i.e., source *S*, relay *R* and destination *D*. The DF relaying technique is considered in the coded cooperative scheme. Two different linear block codes at the source S and relay *R* are denoted by $C_{S}{(n}_{1}{,\;k}_{1}{,\;d}_{1})$ and $C_{R}{(n}_{2}{,\;k}_{2}{,\;d}_{2})$, respectively, where $n_{i}$, $k_{i}$ and $d_{i}$ (i=1, 2) denote the codeword length, code dimension and minimum distance, respectively. Furthermore, two time slots are required to complete an overall transmission.

In the first time slot, the linear block code $C_{S}{(n}_{1}{,\;k}_{1}{,\;d}_{1})$ at the source *S* encodes the information sequence f into the codeword sequence c further given to the modulator. The source broadcasts the generated modulated sequence towards the relay and destination.

In the second time slot, the demodulation at the relay is performed to demodulate the accepted signal and generates the estimated codeword sequence $\bar{c}$ that is fed into the decoder to obtain the estimated information sequence $\bar{f}$ with length $k_{1}$. In our proposed scheme, the information selection block is adopted at the relay. By information selection, $k_{2}$ ($k_{2}{\;<\;k}_{1}$) symbols are chosen from $\bar{f}$ to acquire the information sequence ${\bar{f}}_{j}$ with length $k_{2\;}$, where ${\bar{f}}_{j}$ relies on $\bar{f}$ and j (*j* = 1, 2, …, $C_{k_{1}\;}^{k_{2}}$) represents the order of selections with $C_{k_{1}\;}^{k_{2}}$ denoting the binomial coefficient. The linear block code $C_{R}{\;(n}_{2}{,\;k}_{2}{,\;d}_{2})$ then encodes the sequence ${\bar{f}}_{j}$ into the codeword $c_{j}$. Through the cooperation between the source and relay, the codeword set generated at the destination D is expressed as

(1) $$C_{D}^{\left(j\right)}(n_{1}+n_{2},k_{1})=\{|c|c_{j}|:c\in C_{S}(n_{1},k_{1},d_{1}),c_{j}\in C_{R}(n_{2},k_{2},d_{2})\}$$

where $\left|c\right|c_{j}|\;$ is the series concatenation of c and $c_{j}$. If the code $C_{R}{\;(n}_{2}{,\;k}_{2}{,\;d}_{2})$ has additional information $f^{\prime }$ independent of the sequence $\bar{f}$, $C_{R}{\;(n}_{2}{,\;k}_{2}{,\;d}_{2})$ will generate the codeword $c^{\prime }\in C_{R}\left(n_{2},k_{2},d_{2}\right)$. In this case, the code obtained at the destination D is denoted as follows:

(2) $$C_{D}(n_{1}+n_{2},k_{1})=\{|c|c^{\prime }|:c\in C_{S}(n_{1},k_{1},d_{1}),c^{\prime }\in C_{R}(n_{2},k_{2},d_{2})\}$$

Based on the above analysis, it is noticed that the codeword set $C_{D}^{\left(\;j\;\right)}{(n}_{1}{+n}_{2}{,\;k}_{1})$ produced in the *j*-th selection is a subset of code $C_{D}{(n}_{1}{+n}_{2}{,\;k}_{1})$, i.e., $C_{D}^{\left(\;j\;\right)}{(n}_{1}{+n}_{2}{,\;k}_{1})\;\subseteq {\;C}_{D}{(n}_{1}{+n}_{2}{,\;k}_{1})$.

### 3.2. Incorporation of STBC-SM into Distributed Channel Coding

Coded cooperation is an efficient way to combat channel fading and enlarge network coverage. Moreover, in modern wireless communications, there have been strong demands for superior error performance. Thus, for coded cooperative communication schemes, further improving the system performance becomes urgent.

Many available studies have presented that, by employing the MIMO technology in the cooperative scheme, the system performance is enhanced. Among the existing MIMO schemes, the famous STBC-SM technique not only removes the ICI, but also can take advantage of the transmit diversity. Moreover, it has been reported in [25] that STBC-SM can be easily employed in the DF based cooperative communications. Thus, the STBC-SM technology is considered in our investigated distributed channel coding scheme by subset approach. In this way, the distributed channel coding scheme in conjunction with STBC-SM has a potential to exploit the spatial diversity and cooperation, thus further improving the performance.

As previously stated, the GRS code has many advantages. For instance, it can achieve the singleton bound and correct the maximum number of errors as a special MDS code. Moreover, the GRS code can correct the burst errors and possesses an excellent algebraic structure and extensive applications. Hence, in the distributed coded communication scheme integrated with STBC-SM, the GRS code is utilized to perform error control. We detail the contents in Section 4. Note that, in the following description for the DGRSC-STBC-SM, the codeword lengths at the *S* and *R* are assumed to be the same, i.e., $n_{1}=n_{2}$.

## 4. Distributed GRS-Coded STBC-SM Scheme for Wireless Communications

**Figure 2:**
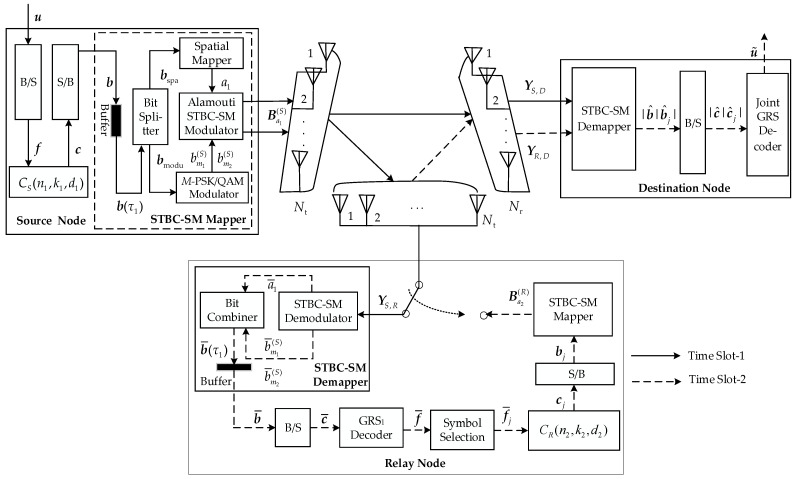
System model of half-duplex DGRSC-STBC-SM scheme.

The DGRSC-STBC-SM scheme is proposed in this section. Figure 2 depicts the system model of the half-duplex DGRSC-STBC-SM scheme, where $N_{t}$, $N_{t}$ and $N_{r}$ antennas are deployed at the source S, relay R and destination *D*, respectively. In the coded cooperative Alamouti STBC-SM, it takes two time slots to complete an overall transmission.

During time slot-1, the source S firstly uses the bits to symbols (B/S) block to convert the binary message bit sequence u into the non-binary symbol sequence ${f=[\;f}_{0}{,\;f}_{1}{,\ldots ,\;f}_{k_{1}-1}]$, where $f_{s}$ is an element of the field $F_{q}$ with ${q=2}^{k}$ and k being a positive natural number. The GRS code $C_{S}{\;(n}_{1}{,\;k}_{1}{,\;d}_{1})$ over $F_{q}$ is used at the source S, and the generator matrix $G_{S}$ has the following form

(3) $$G_{S}=\left[\begin{array}{cccc} v_{0}^{\left(S\right)} & v_{1}^{\left(S\right)} & \cdots & v_{n_{1}-1}^{\left(S\right)}\\ v_{0}^{\left(S\right)}\alpha_{0}^{\left(S\right)} & v_{1}^{\left(S\right)}\alpha_{1}^{\left(S\right)} & \cdots & v_{n_{1}-1}^{\left(S\right)}\alpha_{n_{1}-1}^{\left(S\right)}\\ \vdots & \vdots & \cdots & \vdots \\ v_{0}^{\left(S\right)}{\left(\alpha_{0}^{\left(S\right)}\right)}^{k_{1}-1} & v_{1}^{\left(S\right)}{\left(\alpha_{1}^{\left(S\right)}\right)}^{k_{1}-1} & \cdots & v_{n_{1}-1}^{\left(S\right)}{\left(\alpha_{n_{1}-1}^{\left(S\right)}\right)}^{k_{1}-1} \end{array}\right]$$

where $\alpha_{S}{=[\alpha }_{0}^{\left(S\right)}{,\;\alpha }_{1}^{\left(S\right)}{,\;\ldots ,\;\alpha }_{n_{1}-1}^{\left(S\right)}]$ and $v_{S}{=[v}_{0}^{\left(S\right)}{,\;v}_{1}^{\left(S\right)}{,\;\ldots ,\;v}_{n_{1}-1}^{\left(S\right)}]$ with $\alpha_{l}^{\left(S\right)}\in \;F_{q}$ being distinct elements and ${\;v}_{l}^{\left(S\right)}\;\in \;F_{q}$ being nonzero (but not necessarily distinct) elements. Thus, the information symbol sequence f and the codeword symbol sequence $c\;{=[c}_{0},\;c_{1}{,\;\ldots ,\;c}_{n_{1}-1}]$ are related as follows:

(4) $$c=fG_{S}=[v_{0}^{\left(S\right)}f(\alpha_{0}^{\left(S\right)}),v_{1}^{\left(S\right)}f(\alpha_{1}^{\left(S\right)}),\ldots ,v_{n_{1}-1}^{\left(S\right)}f(\alpha_{n_{1}-1}^{\left(S\right)})]$$

where $c_{l}\;\in \;F_{q}$ and ${f\;(x)=f}_{0}{+f}_{1}{x+\ldots +f}_{k_{1}-1\;}x^{k_{1}-1}$ denotes the polynomial representation of f. Note that the codeword length $n_{1}$ of the GRS code $C_{S}{\;(n}_{1}{,\;k}_{1}{,\;d}_{1})$ is less than q, i.e., $n_{1}\;<\;q$. Since GRS code is an MDS code, its minimum distance is $d_{1}{=n}_{1}{-k}_{1}+1$. For the codeword symbol sequence c, each codeword symbol $c_{l}$ can be represented as the binary vector of length k. After the symbols-to-bits (S/B) converter, we obtain a binary codeword bit sequence b. Through the buffer, the sequence b is further divided into multiple short sequences ${b(\tau }_{1})$ of length k. The length k is mathematically denoted as

(5) $$k=\log_{2}(\varpi )+2\log_{2}(M)=\log_{2}\left({\left\lfloor C_{N_{t}}^{2}\right\rfloor }_{2^{h}}\right)+2\log_{2}(M)$$

where $\tau_{1}=1,\;2,\;\ldots {,\;n}_{1}$, . is the floor operation, $C_{N_{t}}^{2}$ denotes the binomial coefficient, ${\left\lfloor C_{N_{t}}^{2}\right\rfloor }_{2^{h}}$ is an integer power of two with h being a positive integer and M is the modulation order. Based on Equation (5), we have $\varpi M^{2}{=2}^{k}=q$. The sequence ${b(\tau }_{1})$ then enters the bit splitter and is partitioned into $b_{\mathrm{spa}}$ and $b_{\mathrm{modu}}$ with lengths $l_{1}{=\log}_{2}(\varpi )\;$ and $l_{2}{=2\log}_{2}\left(M\right)$, respectively. The spatial mapper receives the sequence $b_{\mathrm{spa}}$ and outputs an active transmit antenna combination (TAC) $a_{1}{=(a}_{1,1}{,\;a}_{1,2})$ out of ϖ TACs, where $a_{1,ο}\;\in {\;\{1,\;2,\;\ldots ,\;N}_{t}\;\}$ (ο=1, 2) is the active transmit antenna index. Similarly, the *M*-PSK/QAM modulator takes the sequence $b_{\mathrm{modu}}$ and generates a pair of modulated symbols ${(b}_{m_{1}}^{\left(S\right)}{,\;b}_{m_{2}}^{\left(S\right)}\;),$ where $b_{m_{\partial }}^{\left(S\right)}\;\in \;\varrho$ with $\varrho {=\{b}_{m_{\partial }}^{\left(S\right)}{,\;m}_{\partial }=1,\;2,\;\ldots ,\;M\;\}$ for ∂=1, 2. This symbol pair ${(b}_{m_{1}}^{\left(S\right)}{,\;b}_{m_{2}}^{\left(S\right)}\;)$ is transmitted through the active TAC $a_{1}$. At the source *S*, the Alamouti STBC-SM modulator outputs the $N_{t}\;\times \;2$ STBC-SM matrix $B_{a_{1}}^{\left(S\right)}$ as

(6) $$B_{a_{1}}^{\left(S\right)}={\left[\begin{array}{ccccccc} 0 & \cdots & b_{m_{1}}^{\left(S\right)}e^{j\vartheta_{a_{1}}} & \cdots & b_{m_{2}}^{\left(S\right)}e^{j\vartheta_{a_{1}}} & \cdots & 0\\ 0 & \cdots & -{\left(b_{m_{2}}^{\left(S\right)}\right)}^{*}e^{j\vartheta_{a_{1}}} & \cdots & {\left(b_{m_{1}}^{\left(S\right)}\right)}^{*}e^{j\vartheta_{a_{1}}} & \cdots & 0 \end{array}\right]}^{T}$$

where ${[.]}^{T}$ and (.)* denote transpose and complex conjugation, respectively, $\vartheta_{a_{1}}$ is the rotation angle for the TAC $a_{1}$ and $B_{a_{1}}^{\left(S\right)}\;\in \;\varphi$ with φ being the set of all $\varpi M^{2}$ transmission matrices. The detailed contents regarding the construction of the STBC-SM codeword, selection of TACs and determination of the optimal rotation angle have been introduced in [5]. Let $\phi_{\varpi }$ be the set of all active TACs $a_{1}$, and $\chi_{\varpi }$ is the set of all $\vartheta_{a_{1}}$. For example, $\phi_{\varpi }=\phi_{4}=\{\left(1,\;2\right),\;\left(3,\;4\right),\;\left(2,\;3\right),\;\left(4,\;1\right)\}$ and $\chi_{\varpi }{=\chi }_{4}=\{0,\;0,\;0.61,\;0.61\}$ for $N_{t}=4$ and 4-QAM. To ease understanding, Table 1 clearly lists the mapping procedure for the STBC-SM with codes over $F_{16}{=\{0,\;1,\;\alpha ,\;\ldots ,\;\alpha }^{14}\}$, where $\alpha \;\in \;F_{16}$ is the root of the primitive polynomial ${1+x+x}^{4}$ over $F_{2}$. The STBC-SM codeword $B_{a_{1}}^{\left(S\right)}$ is transmitted towards the relay node *R* that receives the $N_{t}\;\times \;2$ matrix $Y_{S,R}$ mathematically formulated as:

(7) $$Y_{S,R}=\sqrt{1/2}H_{S,R}B_{a_{1}}^{\left(S\right)}+N_{S,R}$$

where $H_{S,\;R}$ and ${\;N}_{S,\;R}$ are the $N_{t}{\;\times \;N}_{t}$ source-to-relay channel matrix and $N_{t}\;\times \;2$ source-to-relay noise matrix, respectively. The entries of $H_{S,\;R}$ and ${\;N}_{S,\;R}$ separately follow the complex Gaussian distributions CN(0, 1) and $CN\left({0,\;\sigma }^{2}\right)$ with $\sigma^{2}$ being the variance of noise.

During time slot-2, the STBC-SM demodulator performs maximum likelihood detection for received signals and yields the estimated TAC ${\bar{a}}_{1}$ and symbol pair $({\bar{b}}_{m_{1}}^{\left(S\right)},\;{\bar{b}}_{m_{2}}^{\left(S\right)})$ given into the bit combiner to output the sequence ${\bar{b}}_{\left(\tau_{1}\right)}$. Through the buffer, the estimated codeword bit sequence $\bar{b}$ is generated. The B/S block transforms the bit sequence $\bar{b}$ into the non-binary codeword symbol sequence $\bar{c}$. In the following, the *q*-ary GRS_1_ decoder employing Euclidean iterative decoding algorithm [23] decodes the sequence $\bar{c}$ and obtains the estimated information symbol sequence $\bar{f}$. Then, we select $k_{2}$ ($k_{2}{\;<\;k}_{1}$) symbols from $\bar{f}$ to obtain the information symbol sequence ${\bar{f}}_{j}=\left[{\bar{f}}_{0}^{\left(j\right)},{\bar{f}}_{1}^{\left(j\right)},\ldots ,{\bar{f}}_{k_{2}-1}^{\left(j\right)}\right]$, where ${\bar{f}}_{e}^{\left(j\right)}\in {𝔽}_{q}$ and j is the order of the selections indexed by

(8) $$j\in \Lambda =\{1,2,\ldots ,L=C_{k_{1}}^{k_{2}}\}$$

Note that each *j* is one-to-one corresponding to a unique $k_{2}$-dimensional vector, called the selection pattern, to indicate each of the $k_{2}$ symbols selected from $k_{1}$ information symbols and the related position, i.e.,

(9) $$j↔\Omega_{j}=[\gamma_{1}^{\left(j\right)},\gamma_{2}^{\left(j\right)},\ldots ,\gamma_{k_{2}}^{\left(j\right)}],\;\mathrm{where}$$

(10) $$0\leq \gamma_{1}^{\left(j\right)}<\gamma_{2}^{\left(j\right)}<\ldots <\gamma_{k_{2}}^{\left(j\right)}\leq k_{1}-1$$

All the selection patterns form a set given as

(11) $$\psi =\{\Omega_{j}|j\in \Lambda \}=\{\;[\gamma_{1}^{\left(j\right)},\gamma_{2}^{\left(j\right)},\ldots ,\gamma_{k_{2}}^{\left(j\right)}]|0\leq \gamma_{1}^{\left(j\right)}<\gamma_{2}^{\left(j\right)}<\ldots <\gamma_{k_{2}}^{\left(j\right)}\leq k_{1}-1,j\in \Lambda \}$$

Section 5 will introduce the algorithm determining an optimized selection pattern. The relay R employs the GRS code $C_{R}{\;(n}_{2}{,\;k}_{2}{,\;d}_{2})$ with $d_{2}{=n}_{2\;}{-\;k}_{2}+1$ over $F_{q}$ to encode ${\bar{f}}_{j}$. The generator matrix $G_{R}$ is

(12) $$G_{R}=\left[\begin{array}{cccc} v_{0}^{\left(R\right)} & v_{1}^{\left(R\right)} & \cdots & v_{n_{2}-1}^{\left(R\right)}\\ v_{0}^{\left(R\right)}\alpha_{0}^{\left(R\right)} & v_{1}^{\left(R\right)}\alpha_{1}^{\left(R\right)} & \cdots & v_{n_{2}-1}^{\left(R\right)}\alpha_{n_{2}-1}^{\left(R\right)}\\ \vdots & \vdots & \cdots & \vdots \\ v_{0}^{\left(R\right)}{\left(\alpha_{0}^{\left(R\right)}\right)}^{k_{2}-1} & v_{1}^{\left(R\right)}{\left(\alpha_{1}^{\left(R\right)}\right)}^{k_{2}-1} & \cdots & v_{n_{2}-1}^{\left(R\right)}{\left(\alpha_{n_{2}-1}^{\left(R\right)}\right)}^{k_{2}-1} \end{array}\right]$$

where $\alpha_{R}{=[\alpha }_{0}^{\left(R\right)}{,\;\alpha }_{1}^{\left(R\right)}{,\;\ldots ,\;\alpha }_{n_{2}-1}^{\left(R\right)}]$ and $v_{R}{=[v}_{0}^{\left(R\right)}{,\;v}_{1}^{\left(R\right)}{,\;\ldots ,\;v}_{n_{2}-1}^{\left(R\right)}]\;$ with $\alpha_{g}^{\left(R\right)}\;\in \;F_{q}$ being distinct elements and $v_{g}^{\left(R\right)}\;\in \;F_{q}$ being nonzero (but not necessarily distinct) elements. Therefore, the information symbol sequence ${\bar{f}}_{j}$ and the codeword symbol sequence $c_{j}{=[c}_{0}^{\left(j\right)}{,\;c}_{1}^{\left(j\right)}{,\;\ldots ,\;c}_{n_{2}-1}^{\;\left(j\right)}]$ are related as follows:

(13) $$c_{j}={\bar{f}}_{j}G_{R}=\left[v_{0}^{\left(R\right)}{\bar{f}}_{j}\left(\alpha_{0}^{\left(R\right)}\right),v_{1}^{\left(R\right)}{\bar{f}}_{j}\left(\alpha_{1}^{\left(R\right)}\right),\ldots ,v_{n_{2}-1}^{\left(R\right)}{\bar{f}}_{j}\left(\alpha_{n_{2}-1}^{\left(R\right)}\right)\right]$$

where $n_{2}\;<\;q$, $c_{g}^{\;(\;j\;)\;}\in \;F_{q}$ and ${\bar{f}}_{j}\left(x\right)={\bar{f}}_{0}^{\left(j\right)}+{\bar{f}}_{1}^{\left(j\right)}x+\ldots +{\bar{f}}_{k_{2}-1}^{\left(j\right)}x^{k_{2}-1}$ denotes the polynomial representation of ${\bar{f}}_{j}$. Next, the S/B block converts the codeword symbol sequence $c_{j}$ into the codeword bit sequence $b_{j}$ fed into the STBC-SM mapper to obtain the transmission matrix $B_{a_{2}}^{\left(R\;\right)}$ defined like $B_{a_{1}}^{\left(S\right)}$ in Equation (6). Then, the relay *R* sends $B_{a_{2}}^{\left(R\;\right)}$ to *D.*

During the first and second time slots, the destination D receives matrices $Y_{S,\;D}$ and $Y_{R,\;D}$ that are formulated as

(14) $$Y_{S,D}=\sqrt{1/2}H_{S,D}B_{a_{1}}^{\left(S\right)}+N_{S,D}$$

(15) $$Y_{R,D}=\sqrt{1/2}H_{R,D}B_{a_{2}}^{\left(R\right)}+N_{R,D}$$

where $H_{S,\;D}$ and $H_{R,\;D}$ are the $N_{r}{\;\times \;N}_{t}\;$ channel matrices between the destination and source and relay, respectively, and they are defined like $H_{S,\;R}$ in Equation (7). Furthermore, $N_{S,\;D}$ and $N_{R,\;D}$ are the $N_{r}\;\times \;2$ noise matrices between the destination and source and relay, and their definitions are similar to $N_{S,\;R}$ in Equation (7). At the destination, the STBC-SM demapper then performs the demodulation for the received signals. After that, the estimated codeword bit sequence $\left|\hat{b}\right|{\hat{b}}_{j}|$ is generated and further converted into the codeword symbol sequence $\left|\hat{c}\right|{\hat{c}}_{j}|$ through the B/S block. Finally, the designed decoding algorithm is employed to produce the estimated message bit sequence $\tilde{u}$. Section 6 will introduce the contents of the decoding algorithm.

## 5. Proposed Efficient Symbol Selection Algorithms

At the source, $k_{1}$ symbols are encoded by $C_{S}{\;(n}_{1}{,\;k}_{1}{,\;d}_{1})$. The relay needs to select $k_{2}$ symbols from $k_{1}$ symbols for further encoding by $C_{R}{\;(n}_{2}{,\;k}_{2}{,\;d}_{2})$. Through the cooperation between the source and relay, the destination generates a joint codeword set. To acquire the optimized codeword set resulting from the optimized selection, two efficient symbol selection algorithms are proposed. The following algorithms are presented under the assumption that the relay can decode correctly.

### 5.1. Algorithm 1: Optimal Symbol Selection Algorithm

In this subsection, we discuss the proposed optimal symbol selection algorithm. First, we introduce the general description of the algorithm. Then, an example is shown for a better understanding.

#### 5.1.1. General Description of the Optimal Symbol Selection Algorithm

At the relay, it is required to choose $k_{2}$ symbols from $k_{1}$ symbols. Thus, there are $L=C_{k_{1}\;}^{k_{2}}$ selections in total. For the j-th (j∈Λ = {1, 2,…, *L*}) selection, the destination generates the codeword set $C_{D}^{\left(\;j\;\right)}{(n}_{1}{+n}_{2}{,\;k}_{1})$ that is the subset of $C_{D}{(n}_{1}{+n}_{2}{,\;k}_{1})$, as introduced in Section 2. The optimal algorithm aims to find the best selection pattern $\eta^{\left(\mathrm{opt}\right)}$ generating the optimal codeword subset $C_{D}^{\left(\;j\;\right)}{(n}_{1}{+n}_{2}{,\;k}_{1})$ with a larger minimum codeword weight from L selection patterns. Assume that the weight distribution of $C_{D}^{\left(\;j\;\right)}{(n}_{1}{+n}_{2}{,\;k}_{1})$ is expressed as

(16) $$W^{\left(j\right)}(Z)=1+B_{d_{1}}^{\left(j\right)}Z^{d_{1}}+B_{d_{1}+1}^{\left(j\right)}Z^{d_{1}+1}+\ldots +B_{n_{1}+n_{2}}^{\left(j\right)}Z^{n_{1}+n_{2}}$$

In (16), $B_{w}^{\left(\;j\;\right)}$ represents the number of codewords with weight ${\mathrm{wt}(|c|c}_{j}|)=w$ ${(d}_{1}\leq w\;\leq {\;n}_{1}{+n}_{2})$ at the destination. The general steps of the optimal algorithm are as follows:

**Step 1**: Determine the set ψ (shown in Equation (11)) of all selection patterns:

**Step 2:** Consider all possible codeword weights $d_{1}\leq {\mathrm{wt}(|c|c}_{j}|)\;\leq {\;n}_{1}{+n}_{2}$ generated by all $q^{k_{1}}$ source information sequences.

**Step 3**: Initialization: t=0, ${w=d}_{1}$, $\psi_{t}=\psi$ and $\Lambda_{t}=\Lambda$.

**Step 4**: For each $j\;\in {\;\Lambda }_{t}$ related $\Omega_{j}\;\in {\;\psi }_{t}$, we find out the number $B_{w}^{\;(\;j\;)}$ of codewords with weight ${\mathrm{wt}(|c|c}_{j}|)=w$. Choose all the indices j resulting in $\underset{j\;\in {\;\Lambda }_{t}}{\min}{\;B}_{w}^{\;(\;j\;)}$ to constitute the subset $\Lambda_{t+1}\;\subseteq {\;\Lambda }_{t\;}$. Find out the subset $\psi_{t+1}\;\subseteq {\;\psi }_{t}$ that corresponds to $\Lambda_{t+1}$.

**Step 5**: Check w and ${|\psi }_{t+1}|$,

(1) If ${w\;<\;n}_{1}{+n}_{2}$ and ${|\psi }_{t+1}|\;\neq \;1$, t →t+1, w →w+1 and return back to step 4.
(2) Else, i.e., ${w=n}_{1}{+n}_{2}$ or ${|\psi }_{t+1}|=1$, the searching algorithm halts.

* Note: In (2), if ${|\psi }_{t+1}|=1$, the only element of $\psi_{t+1}$ is determined as the optimal selection pattern $\eta^{\left(\mathrm{opt}\right)}$; if ${|\psi }_{t+1}|\;\neq \;1,$ take any element of $\psi_{t+1}$ as the optimal selection pattern $\eta^{\left(\mathrm{opt}\right)}$.

#### 5.1.2. Example 1

To better understand the optimal symbol selection algorithm, an example is then presented. At the source *S*, the GRS code is $C_{S}{\;(n}_{1}{,\;k}_{1}{,\;d}_{1}{)=C}_{S}\;(5,\;3,\;3)$. Furthermore,

(17) $$\alpha_{S}=[\beta ,\beta^{2},\beta^{3},\beta^{4},\beta^{5}]$$

(18) $$v_{S}=[\beta ,\beta^{2},\beta^{3},\beta^{4},\beta^{4}]$$

over $F_{8}=\{0,\;1,\;\beta ,\;\ldots ,\;\beta^{6}$} are used, where $\beta \in \;F_{8}$ is the root of primitive polynomial ${1+x+x}^{3}$ over $F_{2}$. At the relay R, the GRS code $C_{R}{\;(n}_{2}{,\;k}_{2}{,\;d}_{2}{)=C}_{R}\;(5,\;2,\;4)$ is used, and

(19) $$\alpha_{R}=[1,\beta ,\beta^{2},\beta^{4},\beta^{6}]$$

(20) $$v_{R}=[1,\beta ,\beta^{2},\beta^{3},\beta^{5}]$$

over $F_{8}$ are considered. The generation process of the optimal pattern $\eta^{\left(\mathrm{opt}\right)}$ is as follows:

**Step1:** Determine the set ψ of all selection patterns as

(21) $$\psi =\{\Omega_{j}|j\in \Lambda \}=\{[0,1],[0,2],[1,2]\}$$

**Step 2:** Determine all possible codeword weights ${\mathrm{wt}(|c|c}_{j}\left|\right)$ at the *D* generated by 512 source information sequences, as shown in Table 2.

**Step 3:** For each j ∈ Λ related $\Omega_{j}\;\in \;\psi$, find out the number $B_{3}^{\left(\;j\;\right)}$ of codeword weight ${\mathrm{wt}(|c|c}_{j}|)=3$, as shown in Table 3. Select all the indices j which result in $\underset{j\;\in \;\Lambda }{\min}B_{3}^{\left(\;j\;\right)}$ to form the set $\Lambda_{1}=\{1,\;2,\;3\}$. Find out $\psi_{1}$={$\Omega_{1}{,\;\Omega }_{2}{,\;\Omega }_{3}\}$ corresponding to $\Lambda_{1}$. Since ${|\psi }_{1}|=3\;>1$, we proceed to Step 4.

**Step 4:** For each $j\;\in {\;\Lambda }_{1}$ related $\Omega_{j}\;\in {\;\psi }_{1}$, find out the number $B_{4}^{\left(\;j\;\right)}$. Select all the indices j resulting in $\underset{j\;\in {\;\Lambda }_{1}}{\min}B_{4}^{\left(\;j\;\right)}$ to constitute the set $\Lambda_{2}=\{1,\;2,\;3\}$ and then take the set $\psi_{2}$=${\{\Omega }_{1}{,\;\Omega }_{2}{,\;\Omega }_{3}\}$ corresponding to $\Lambda_{2}$. Since ${|\psi }_{2}|=3>\;1$, we proceed to Step 5.

**Step 5**: For each $j\;\in {\;\Lambda }_{2}$ related $\Omega_{j}\;\in {\;\psi }_{2}$, find out the number $B_{5}^{\left(\;j\;\right)}$. Select all the indices j resulting in $\underset{j\;\in {\;\Lambda }_{2}}{\min}B_{5}^{\left(\;j\;\right)}$ to constitute the set $\Lambda_{3}=\{1,\;2,\;3\}$ and then take the set $\psi_{3}$=${\{\Omega }_{1}{,\;\Omega }_{2}{,\;\Omega }_{3}\}$ corresponding to $\Lambda_{3}$. Since ${|\psi }_{3}|=3>\;1$, we proceed to Step 6.

**Step 6**: Similar to the above steps, we get $\psi_{4}$=${\{\Omega }_{2}\}$. Since ${|\psi }_{4}|=1$, the search algorithm is terminated.

The determined optimal selection pattern $\eta^{\left(\mathrm{opt}\right)}{=\Omega }_{2}\;=[0,\;2]\;$ is fixed at the relay, and the $k_{2}=2$ symbols dictated by $\Omega_{2}$ are encoded by $C_{R}\;(5,\;2,\;4)$. Accordingly, the codeword subset $C_{D}^{\left(2\right)}\left(10,\;3\right)$ of $C_{D}\left(10,\;3\right)$ is generated at the destination. From Table 3, it is seen that our algorithm may not improve the minimum codeword weight. However, the number of low-weight ${\mathrm{wt}(|c|c}_{j}|)=7$ generated by the determined pattern $\eta^{\left(\mathrm{opt}\right)}{=\Omega }_{2}\;=[0,\;2]\;$ is less than that generated by the other two patterns. Thus, our algorithm avoids the bad scenarios that a large number of low-weight codewords are results at the destination.

### 5.2. **Algorithm 2:** Low-Complexity Symbol Selection Algorithm

In the optimal **Algorithm 1**, all the $q^{{\;k}_{1}}$ information sequences at the source are considered to search the best selection pattern from all L selection patterns. For a larger block length code, the search complexity is very high. Thus, we propose the symbol selection algorithm (**Algorithm 2**) with a reduced complexity. Similar to **Algorithm 1**, we introduce **Algorithm 2** by the general description and an example.

#### 5.2.1. General Description of the Low-Complexity Symbol Selection Algorithm

**Algorithm 2** has two differences compared to **Algorithm 1**. The first point is that partial ($k_{b}$) information symbol sequences are considered at the source, where $k_{b}{\;<\;q}^{{\;k}_{1}}$. The second point is that partial (*P*) selection patterns are considered at the relay, where *P* < *L*. Thus, in **Algorithm 2**, ${\;k}_{b}$ information symbol sequences are considered for determining the pattern $\eta^{\left(\mathrm{low}\right)}$ from *P* patterns. It effectively converts an exhaustive search algorithm into a partial search algorithm. For the j-th (j = 1,2, …, *P*) selection, the destination generates the subset $C_{D}^{\left(\;j\;\right)}{(n}_{1}{+n}_{2}{,\;k}_{1})$ of $C_{D}{(n}_{1}{+n}_{2}{,\;k}_{1})$. The specific steps of **Algorithm 2** are as follows.

**Step 1:** Determine $k_{b}$ information symbol sequences  f.

From Equation (4), we see for wt(c)=i ($d_{1}\leq \;i\;\leq {\;n}_{1}$), f(x) has *n*${=n}_{1}-\;i$ various roots in $\alpha_{S}$ of length $n_{1}$. To reasonably obtain the partial information symbol sequences f at the source, the following method is used:

(1) Split $F_{q}$ into two parts, where all the ${\;n}_{1}$ elements of $\alpha_{S}$ form the first part, and the other ${q\;-\;n}_{1}$ elements in $F_{q}$ but not in $\alpha_{S}$ form the second part.
(2) J (n ≤ J ≤deg(f (x))) elements are reasonably selected from $F_{q}$ as all roots of f(x). On the one hand, *n* elements of J elements are randomly chosen in the first part, and the remaining J − n elements are fixedly selected in the second part, which generates $C_{n_{1}}^{n}$ cases. On the other hand, the roles of random and fixed selection are reversed, i.e., we randomly choose J − n elements from the second part, and fixedly choose the n elements from the first part, which yields $C_{{q-n}_{1}}^{J-\;n}$ cases.
(3) Based on the above process, $k_{b}$ information symbol sequences  f are obtained.

**Step 2:** Determine the set $\bar{𝜓}$ of *P* selection patterns.

**Figure 3:**
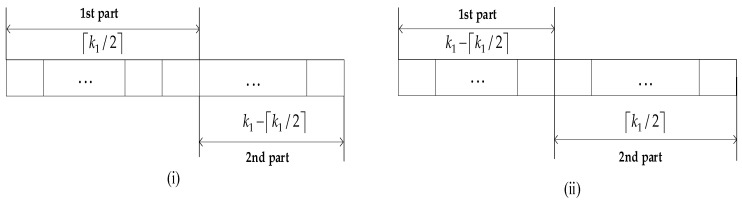
Symmetric structures of $k_{1}$ symbols (**i**) more positions are in the first part (**ii**) more positions are in the second part.

(1) First partition ${\;k}_{1}$ information symbols at the source into two parts. Scenario (i): the first $\left[{\;k}_{1}/2\right]$ symbols and the last ${\;k}_{1}-[{\;k}_{1}/2]$ symbols form the first and second parts, respectively, where $\left[{\;k}_{1}/2\right]$ is the smallest integer larger than or equal to $\left[{\;k}_{1}/2\right]$. Scenario (ii): the first ${\;k}_{1}-[{\;k}_{1}/2]$ symbols and the last $\left[{\;k}_{1}/2\right]$ symbols form the first and second parts, respectively. The symmetric structures of $k_{1}$ symbols are shown in Figure 3.
(2) The relay selects ${\;k}_{2}$ symbols from ${\;k}_{1}$ symbols. In scenario (i), we select more symbols in the first part. Specifically, $\left([k_{2}/2\;]\leq \;\Gamma \;\leq {\;\min\;(\;k}_{2},[{\;k}_{1}/2)\right]$ symbols are randomly chosen in the first part and ${\;k}_{2}-\;\Gamma$ symbols are fixedly chosen in the second part, which generates $C_{\left[{\;k}_{1}/2\right]}^{\;\Gamma }$ cases. In scenario (ii), more symbols are chosen in the second part. Specifically, randomly choose Γ symbols in the second part and fixedly choose ${\;k}_{2}-\;\Gamma$ symbols in the first part, which also generates $C_{\left[{\;k}_{1}/2\right]}^{\;\Gamma }$ cases.
(3) Obtain the set $\bar{𝜓}$ of *P* selection patterns by the above process.

**Step 3:** Take the selection index set $\bar{\Lambda }=\left\{1,2,\ldots ,\left|\bar{\psi }\right|\right\}$ corresponding to the set $\bar{𝜓}$.

**Step 4:** Other steps refer to the steps 2–5 of **Algorithm 1**. Finally, we have the optimized selection pattern $\eta^{\left(\mathrm{low}\right)}$ from *P* selection patterns.

#### 5.2.2. Example 2

At the source *S*, $C_{S}{\;(n}_{1}{,\;k}_{1}{,\;d}_{1}{)=C}_{S}\;(10,\;5,\;6).\;$ The parameter vectors

(22) $$\alpha_{S}=[\alpha ,\alpha^{2},\alpha^{3},\alpha^{4},\alpha^{5},\alpha^{6},\alpha^{8},\alpha^{9},\alpha^{12},\alpha^{7}]$$

(23) $$v_{S}=[\alpha ,\alpha^{2},\alpha^{3},\alpha^{4},\alpha^{4},\alpha^{5},\alpha^{8},\alpha^{7},\alpha^{10},\alpha^{10}]$$

over $F_{16}{=\{0,\;1,\;\alpha ,\;\ldots ,\;\alpha }^{14}\}$ are used, where $\alpha \;\in \;F_{16}$ is the root of primitive polynomial ${1+x+x}^{4}$ over $F_{2}$. At the relay R, we use $C_{R}{\;(n}_{2}{,\;k}_{2}{,\;d}_{2}{)=C}_{R}\;(10,\;3,\;8)$ with

(24) $$\alpha_{R}=[1,\alpha ,\alpha^{2},\alpha^{4},\alpha^{6},\alpha^{5},\alpha^{7},\alpha^{9},\alpha^{10},\alpha^{11}]$$

(25) $$v_{R}=[1,\alpha ,\alpha^{2},\alpha^{3},\alpha^{5},\alpha^{5},\alpha^{7},\alpha^{5},\alpha^{8},\alpha^{9}]$$

over $F_{16}$. The generation process of the low complexity pattern $\eta^{\left(\mathrm{opt}\right)}$ is as follows:

**Step 1:** Determine $k_{b}$ information symbol sequences  f.

(1) Firstly divide 16 elements of $F_{16}$ into two parts, where all the 10 elements of $\alpha_{s}$ constitute the first part and the six elements in $F_{16}$ but not in $\alpha_{s}$ constitute the second part.
(2) Select J (10 − i ≤ J ≤deg(f(x))=4) elements from $F_{q}$ as all roots of f(x). (i) 10 − i  elements of J elements are randomly selected from the first part and the remaining J −(10 − i) elements are fixedly selected from the second part. (ii) J − (10 − i) elements of J elements are randomly selected from the second part and the remaining 10 − i elements are fixedly selected from the first part. The process of choosing J elements is listed in Table 4.
(3) Obtain $k_{b}=6440$ information symbol sequences  f based on (1) and (2).

**Step 2:** Determine the set $\bar{𝜓}$ of *P* selection patterns.

(1) Divide ${\;k}_{1}=5$ information symbols at the source into two parts. Scenario (i): the first three symbols and the last two symbols form the first and second parts, respectively. Scenario (ii): the first two symbols and the last three symbols form the first and second parts, respectively.
(2) The relay selects ${\;k}_{2}=3$ symbols from ${\;k}_{1}=5$ symbols. In scenario (i), we randomly select two symbols in the first part and fixedly select one symbol in the second part. In scenario (ii), we randomly select two symbols in the second part and fixedly select one symbol in the first part.
(3) The set of selection patterns is determined as
  (26) $$\begin{array}{cc} \bar{\psi } & =\{\Omega_{1}{,\;\Omega }_{2}{,\;\Omega }_{3}{,\;\Omega }_{4}{,\;\Omega }_{5}{,\;\Omega }_{6}{,\;\Omega }_{7}\}\\ & \;=\{[0,\;1,\;3],\;[0,\;2,\;3],\;[1,\;2,\;3],\;[0,\;1,\;2],\;[1,\;2,\;4],\;[1,\;3,\;4],\;[2,\;3,\;4]\} \end{array}$$

**Step 3:** Obtain the selection index set ${\bar{\Lambda }}_{1}=\left\{1,2,\ldots ,|\bar{\psi }|=7\right\}$.

**Step 4**: Determine all possible codeword weights ${\mathrm{wt}(|c|c}_{j}\left|\right)$ at the *D* generated by 6440 source information sequences, as shown in Table 5.

**Step 5:** For each $j\in \bar{\Lambda }$ related $\Omega_{j}\in \bar{𝜓}$, find out the number ${\bar{B}}_{6}^{\left(j\right)}$ of codeword weight ${\mathrm{wt}(|c|c}_{j}|)=6$, as shown in Table 6. Select all the indices j which result in $\underset{j\;\in \;\Lambda }{\min}B_{6}^{\left(\;j\;\right)}$ to form the set ${\bar{\Lambda }}_{1}=\left\{1,2,\ldots ,7\right\}$. Find out ${\bar{\psi }}_{1}=\left\{\Omega_{1},\Omega_{2},\ldots ,\Omega_{7}\right\}$ corresponding to ${\bar{\Lambda }}_{1}$. Since $\left|{\bar{\psi }}_{1}\right|=7>1$, the search algorithm proceeds to Step 6.

**Step 6:** Determine the set ${\bar{\Lambda }}_{2}=\left\{1,2,\ldots ,7\right\}$ and ${\bar{\psi }}_{2}=\left\{\Omega_{1},\Omega_{2},\ldots ,\Omega_{7}\right\}$. Since $\left|{\bar{\psi }}_{2}\right|=7>1$, the search algorithm proceeds to Step 7.

**Step 7:** Determine the set ${\bar{\Lambda }}_{3}=\left\{1,3,4,5,6,7\right\}$ and ${\bar{\psi }}_{3}=\{\Omega_{1}{,\;\Omega }_{3}{,\;\Omega }_{4}{,\;\Omega }_{5}{,\Omega }_{6},{\;\Omega }_{7}\}$. Since $\left|{\bar{\psi }}_{3}\right|=6>1$, the search proceeds to Step 8.

**Step 8:** Determine the set ${\bar{\Lambda }}_{4}=\left\{6\right\}$ and ${\bar{\psi }}_{3}=\left\{\Omega_{6}\right\}$. Since $\left|{\bar{\psi }}_{4}\right|=1$, the search algorithm is terminated. The unique selection pattern is determined as $\eta^{\left(\mathrm{low}\right)}{=\Omega }_{6}=[1,\;3,\;4]$.

The determined optimized selection pattern $\eta^{\left(\mathrm{low}\right)}{=\Omega }_{6}=[1,\;3,\;4]$ is fixed at the relay, and the $k_{2}=3$ symbols dictated by $\Omega_{6}$ are encoded by $C_{R}\;(10,\;3,\;8)$. Accordingly, the destination generates the codeword subset $C_{D}^{\left(6\right)}\left(20,\;5\right)$ of $C_{D}\left(20,\;5\right)$.

### 5.3. Complexity Comparisons between Two Algorithms

The complexity of the proposed two algorithms is calculated in terms of the addition and multiplication operations. At the source *S*, $\zeta_{S}^{\times }{=n}_{1}k_{1}$ multiplication operations and $\zeta_{S}^{+}{=n}_{1}{(k}_{1\;}-1)$ addition operations are required to encode an information sequence with length $k_{1}$. Thus, there are $\zeta_{S}{=\zeta }_{S}^{\times }{+\zeta }_{S}^{+}{=n}_{1}{(2k}_{1}-1)$ elementary operations involved in encoding one information sequence at the source. Similarly, $\zeta_{R}{=\zeta }_{R}^{\times }{+\zeta }_{R}^{+}{=n}_{2}{(2k}_{2}-1)$ elementary operations are needed to encode one information sequence of length $k_{2}$ at the relay R, where ${\;\zeta }_{R}^{\times }{=n}_{2}k_{2}$ and ${\;\zeta }_{R}^{+}{=n}_{2}{(k}_{2}-1)$.

In **Algorithm 1**, all ${\;q}^{k_{1}}$ information symbol sequences at the source are considered. Assume that, for finding out the number $B_{w}^{\;(\;j\;)}$ of codewords ${|c|c}_{j}|$ with weight ${\mathrm{wt}(|c|c}_{j}{|)=w\;(d}_{1}\leq w\;\leq {\;n}_{1}{+n}_{2})$ at the destination, the considered number of selection patterns (in the relay) is denoted as $N_{{\;R,\;d}_{1}}^{\;(\mathrm{opt})}$, $N_{{\;R,\;d}_{1}+1}^{\;(\mathrm{opt})}$, …, $N_{{R,\;n}_{1}{+n}_{2}}^{\;(\mathrm{opt})}$, respectively. If the best pattern can be determined in finding out the number of codewords ${|c|c}_{j}|$ with weight ${\mathrm{wt}(|c|c}_{j}{|)=w}_{D}^{\left(\mathrm{opt}\right)}$, the optimal symbol selection algorithm will terminate. The overall computational complexity is expressed as follows:

(27) $$\delta^{\left(opt\right)}=q^{k_{1}}\zeta_{S}+\delta_{d_{1}}^{\left(opt\right)}+\delta_{d_{1}+1}^{\left(opt\right)}+\ldots +\delta_{w_{D}^{\left(opt\right)}}^{\left(opt\right)}$$

where $\delta_{\;\nu }^{\left(\mathrm{opt}\right)\;}{=q}^{k_{1}}N_{\;R,\;\nu }^{\;(\mathrm{opt})}\zeta_{R}$ is the complexity for finding each $N_{\;R,\;\nu }^{\;(\mathrm{opt})}$ at the relay.

Now we take into account the computational complexity of **Algorithm 2**. In the algorithm, we consider ${\;k}_{b}$ information symbol sequences at the source. Provided that the pattern is determined when finding out the number of codewords ${|c|c}_{j}|$ with weight ${\mathrm{wt}(|c|c}_{j}{|)=w}_{D}^{\left(\mathrm{low}\right)}$. Then, the complexity of **Algorithm 2** is

(28) $$\delta^{\left(low\right)}=k_{b}\zeta_{S}+\delta_{d_{1}}^{\left(low\right)}+\delta_{d_{1}+1}^{\left(low\right)}+\ldots +\delta_{w_{D}^{\left(low\right)}}^{\left(low\right)}$$

where $\delta_{\;\nu }^{\left(\mathrm{low}\right)}{=k}_{b}N_{\;R,\;\nu }^{\left(\mathrm{low}\right)}\zeta_{R}$ with $N_{\;R,\;\nu }^{\;\left(\mathrm{low}\right)}$ being the considered number of selected patterns for finding out the number ${\bar{B}}_{w}^{\left(j\right)}$ of codewords ${|c|c}_{j}|$ with weight ${\mathrm{wt}(|c|c}_{j}|)=w$ at the destination.

By Equation (27) and Equation (28), we can calculate the complexity of **Algorithm 1** and **Algorithm 2**, respectively. Table 7 shows that the complexity comparison of the two algo-rithms for different coding configurations (which are used in the above two examples). It can be clearly observed from Table 7 that the computational complexity of **Algorithm 2** is significantly decreased over **Algorithm 1**, which well reflects the low-complexity feature of **Algorithm 2**.

## 6. Decoding Algorithm at the Destination

**Step 1:** The destination node takes the first part $\hat{c}$ and the second part ${\hat{c}}_{j}$ of the demodulated codeword symbol sequence $\left|\hat{c}\right|{\hat{c}}_{j}|$ as input, and then uses the *q*-ay GRS_1_ and GRS_2_ decoders employing the Euclidean iterative decoding algorithm to decode them, respectively.

**Step 2:** After their respective decoding of the two GRS decoders, the estimated non-binary message symbol sequences $\hat{f}$ with length $k_{1}$, and ${\hat{f}}_{i}$ with length $k_{2}$ are generated. Then, $\hat{f}$ and ${\hat{f}}_{i}$ are given into the combiner block.

**Step 3:** Select an appropriate threshold ρ. The threshold is the signal-to-noise ratio (SNR) where the bit error rate (BER) performances of $C_{S}{\;(n}_{1}{,\;k}_{1}{,\;d}_{1})$ and $C_{R}{\;(n}_{2}{,\;k}_{2}{,\;d}_{2})$ codes cross each other. By pre-simulating these two signals, this threshold ρ is obtained.

**Step 4:** If SNR ≤ ρ, the output of the combiner block is $\tilde{f}=\hat{f}$. If SNR > ρ, the $k_{2}$ symbols (placed in the selected $k_{2}$ positions) of the non-binary sequence $\hat{f}$ are replaced with the symbol sequence ${\hat{f}}_{j}$, and the joint output $\tilde{f}$ (the update of $\hat{f}$) is yielded by the combiner.

**Step 5:** After the symbols to bits (S/B) block, the non-binary symbol sequence $\tilde{f}$ from the combiner block is transformed into the binary bit sequence $\tilde{u}$ utilized as the estimate of the binary information bit sequence u transmitted by the source node.

This reason for Step 4: because of the larger minimum distance of $C_{R}{\;(n}_{2}{,\;k}_{2}{,\;d}_{2})$ at the relay over $C_{S}{\;(n}_{1}{,\;k}_{1}{,\;d}_{1})$ at the source, those selected $k_{2}$ symbols in $\hat{f}$ are more credible than all those $k_{2}$ symbols in ${\tilde{f}}_{j}$ at SNR ≤ ρ. However, at SNR > ρ, all the $k_{2}$ symbols of ${\tilde{f}}_{j}$ have more reliability than those selected $k_{2}$ symbols in $\hat{f}$.

## 7. Simulation Results

The simulated results are presented to perform evaluation for the BER performance of the proposed DGRSC-STBC-SM scheme and the reference schemes. The slow Rayleigh fading channel, maximum likelihood (ML) detection and Euclidean iterative decoding algorithm are used in the simulations. To better generalize and analyze our investigated schemes, three distributed GRS codes are considered. In the first case, we adopt the GRS codes $C_{S}\;(10,\;5,\;6)$ and $C_{R}\;(10,\;3,\;8)$ over $F_{16}$. In the second case, the GRS codes $C_{S}\;(25,\;19,\;7)$ and $C_{R}\;(25,\;10,\;16)$ over $F_{32}$ are used. In addition, the GRS codes $C_{S}\;(63,\;51,\;13)$ and $C_{R}\;(63,\;31,\;33)$ over $F_{64}$ are employed in the third case. In the first, second and third cases, the code rates are 1/4, 19/50 and 51/126, respectively. The finite fields $F_{16}$,$\;F_{32}$ and $F_{64}$ are constructed using the polynomials ${1+x+x}^{4}$, ${1+x}^{2}{+x}^{5}$ and ${1+x+x}^{6}$, respectively, with α, γ and χ being the roots of ${1+x+x}^{4}$, ${1+x}^{2}{+x}^{5}$ and ${1+x+x}^{6}$, respectively. Table 8 shows the parameter vectors $\alpha_{S}$, $v_{S}$, $\alpha_{R}$ and $v_{R}$ corresponding to the three cases. The SNR of source-to-destination, source-to-relay, and relay-to-destination links are denoted by $\lambda_{S,\;D\;}$, $\lambda_{S,\;R}$ and $\lambda_{R,\;D}$, respectively. Furthermore, the condition, i.e., $\lambda_{R,\;D}{=\lambda }_{S,\;D\;}+2$ is assumed in the proposed DGRSC-STBC-SM scheme. This section depicts the BER performance versus SNR ($\lambda_{S,\;D}$) for all the investigated schemes. Moreover, let all the corresponding receivers possess perfect channel knowledge. In addition, we list the parameters utilized in the simulation, as shown in Table 9.

### 7.1. Performance Comparisons of DGRSC-STBC-SM Scheme under Various Symbol Selection Algorithms

This subsection discusses the performance of the DGRSC-STBC-SM scheme using the proposed symbol selection algorithms and random selection in order to demonstrate the effectiveness of our proposed algorithms.

**Figure 4:**
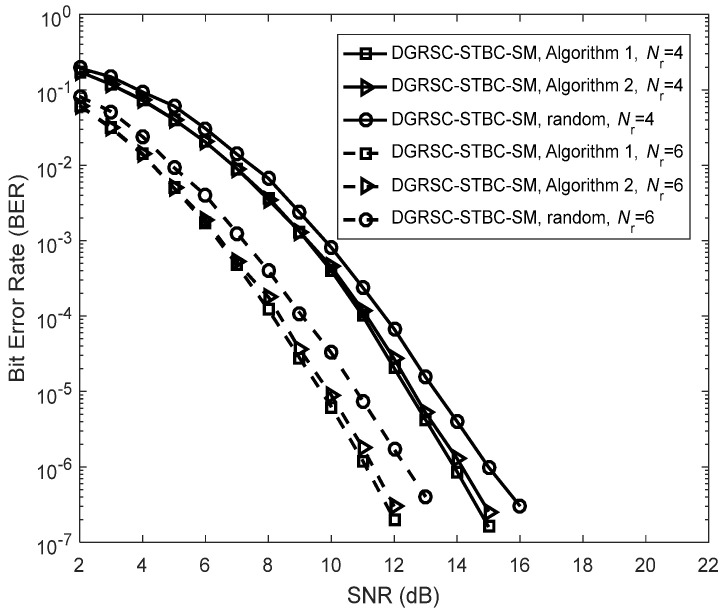
Error performance for DGRSC−STBC−SM using $C_{S}\;(10,\;5,\;6)$ and $C_{R}\;(10,\;3,\;8)$ under different selection algorithms, $N_{t}=4$ and BPSK.

We first discuss the error performance of the DGRSC-STBC-SM under our proposed symbol selection algorithms, i.e., **Algorithm 1** and **Algorithm 2** for the first case, as depicted in Figure 4. The corresponding selection patterns $\eta^{\left(\mathrm{opt}\right)}$ and $\eta^{\left(\mathrm{low}\right)}$ are shown in Table 10. Additionally, we investigate the error performance of the DGRSC-STBC-SM scheme with a random selection pattern for a fair comparison. The ideal source-to-relay channel, i.e., $\lambda_{S,\;R}=\infty$ is supposed. The simulated results of Figure 4 show that when $N_{r}$ is identical, at low SNR the performance of the DGRSC-STBC-SM scheme under **Algorithm 2** approaches that of the DGRSC-STBC-SM scheme under **Algorithm 1**, and their performance difference can be negligible at high SNR. This is because **Algorithm 1** and **Algorithm 2** generate the same minimum codeword weight (i.e., 9) at the destination. It well illustrates that **Algorithm 2** is capable of achieving the balance between the complexity and performance, which reveals the effectiveness of **Algorithm 2**. Moreover, from Figure 4, it is also seen that the error performance advantage of the DGRSC-STBC-SM with **Algorithm 1** and **Algorithm 2** over the random selection pattern under identical conditions. It is because our proposed algorithms increase the minimum codeword weight at the destination, which implies that the proper selection in the relay has a key role in improving the performance.

**Figure 5:**
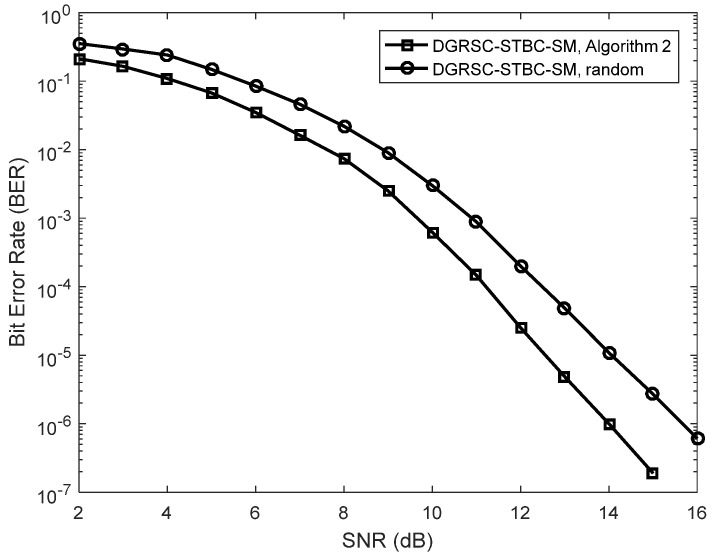
Error performance for DGRSC−STBC−SM using $C_{S}\;(25,\;19,\;7)$ and $C_{R}\;(25,\;10,\;16)$ under different selection algorithms, $N_{t}=3$, $N_{r}=4$ and 4−QAM.

**Figure 6:**
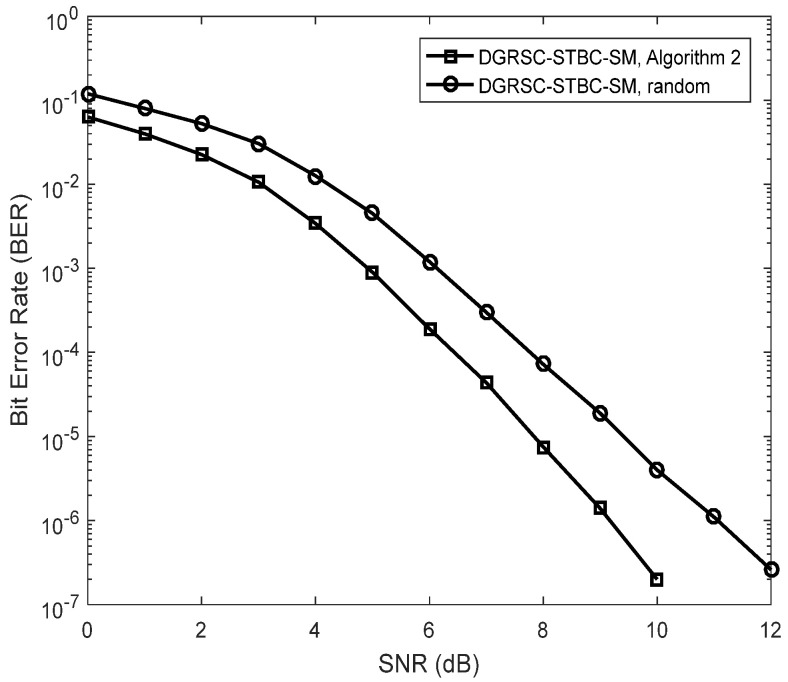
Error performance for DGRSC−STBC−SM using $C_{S}\;(63,\;51,\;13)$ and $C_{R}\;(63,\;31,\;33)$ under different selection algorithms, $N_{t}{=N}_{r}=4$ and 4−QAM.

Due to the effectiveness of **Algorithm 2**, we mainly focus on investigating the performance of the DGRSC-STBC-SM ($\lambda_{S,\;R}=\infty$) under **Algorithm 2**, for the second and third cases with large information sequence length, where the selection patterns $\eta^{\left(\mathrm{low}\right)}$ of the second and third cases are exhibited in Table 10. Figure 5 and Figure 6 show that the DGRSC-STBC-SM scheme having **Algorithm 2** exhibits better performance over the DGRSC-STBC-SM scheme having the random selection pattern. This is because, by adopting the proposed optimized **Algorithm 2**, the codeword set generated at the destination has a larger minimum codeword weight (i.e., 30 for the second case and 50 for the third case).

### 7.2. Error Performance of DGRSC-STBC-SM and Non-Cooperative Counterpart

**Figure 7:**
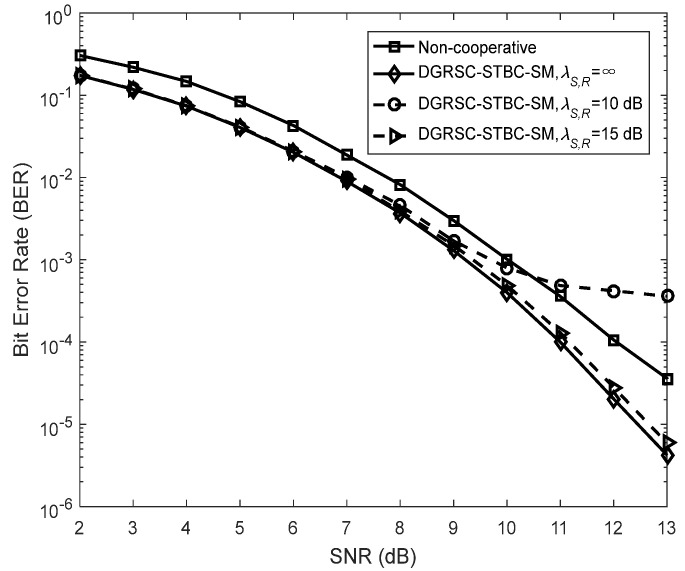
Comparisons between DGRSC−STBC−SM employing $C_{S}\;(10,\;5,\;6)$ and $C_{R}\;(10,\;3,\;8)$ and non−cooperative counterpart under **Algorithm 1**, $N_{t}{=N}_{r}=4$ and BPSK.

**Figure 8:**
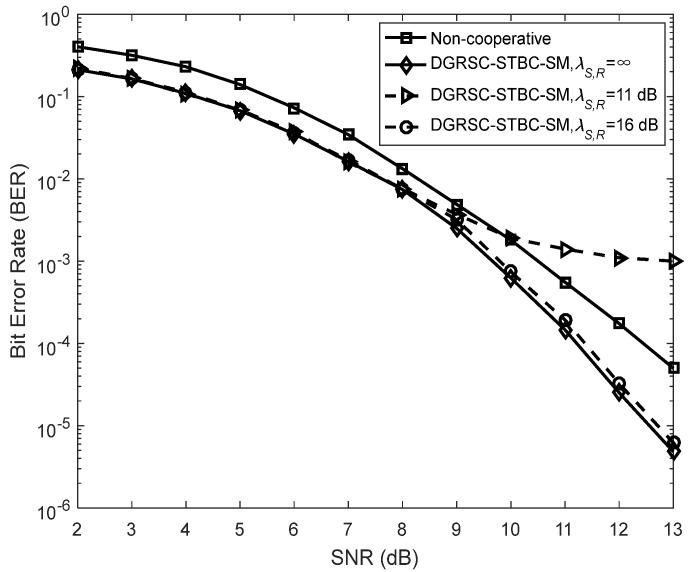
Comparisons between DGRSC−STBC−SM with $C_{S}\;(25,\;19,\;7)$ and $C_{R}\;(25,\;10,\;16)$ and non−cooperative counterpart under **Algorithm 2**, $N_{t}=3$, $N_{r}=4$ and 4−QAM.

**Figure 9:**
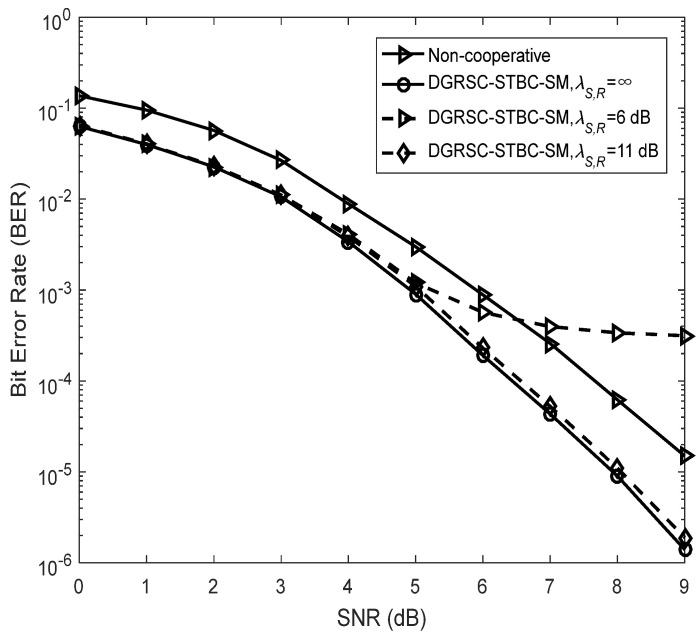
Comparisons between DGRSC−STBC−SM employing $C_{S}\;(63,\;51,\;13)$ *and* $C_{R}\;(63,\;31,\;33)$ and non−cooperative counterpart under **Algorithm 2**,${\;N}_{t}{=N}_{r}=4$ and 4−QAM.

To illustrate the effectiveness of our proposed DGRSC-STBC-SM scheme in cooperative scenarios, we analyze their performance of the DGRSC-STBC-SM and non-cooperative GRS-coded STBC-SM schemes for three cases under an identical code rate. Note that the non-cooperative GRS-coded STBC-SM scheme corresponds to the ideal ($\lambda_{S,\;R}=\infty$) DGRSC-STBC-SM scheme that the relay-to-destination link has no SNR gain over the source-to-destination link, that is to say, $\lambda_{S,\;R}=\infty$ and $\lambda_{R,\;D}{=\lambda }_{S,\;D\;}$ in the non-cooperative scheme. Furthermore, the source and relay nodes in the non-cooperative scheme adopt the coding parameters shown in Table 9, where the source coding and relay coding have a relationship, i.e., the information symbols at the relay are taken from those at the source. Observe from Figure 7, Figure 8 and Figure 9 that, compared with its corresponding non-cooperative counterpart, the DGRSC-STBC-SM scheme ($\lambda_{S,\;R}=\infty$ and $\lambda_{R,\;D}{=\lambda }_{S,\;D\;}+2$) shows better BER performance, where the non-cooperative counterpart corresponds to the DGRSC-STBC-SM with $\lambda_{R,\;D}{=\lambda }_{S,\;D\;}$. This is mainly because the relay node is closer to the destination node than the source node, which helps the correct estimation of the information sequence from the source node.

We also analyze the error performance under the non-ideal source-to-relay channel to better see the impact of practical channel scenarios. The results reveal that if the link between source and relay is the non-ideal ($\lambda_{S,\;R}\;\neq \;\infty$) link with a larger SNR (i.e., 15 dB, 16 dB and 11 dB), as shown in Figure 7, Figure 8 and Figure 9, respectively, the corresponding error performance is very approximate to that of the ideal DGRSC-STBC-SM scheme. However, if the link between source and relay has a low SNR, i.e., 10 dB, 11 dB and 6 dB, the error performance of the DGRSC-STBC-SM scheme becomes worse, and the error floor is generated at the BER $\;\approx \;4{.2\;\times \;10}^{\;-4}$, $1{.4\;\times \;10}^{-\;3}$ and $3{.3\;\times \;10}^{\;-4}$, as exhibited in Figure 7, Figure 8 and Figure 9, respectively. The reason for the error floor phenomenon is that the incorrect decoding in the relay brings the error propagation to the common destination. With the aid of cyclic redundancy check (CRC) technology, the error propagation can be further mitigated, and the error performance will show better improvement. Since the detailed contents are beyond the scope of our research, we do not discuss them in this manuscript.

### 7.3. Performance Comparisons between DGRSC-STBC-SM Scheme and Existing Scheme

To confirm the superiority of the proposed DGRSC-STBC-SM scheme, we perform the performance comparison between the proposed scheme and the existing schemes.

**Figure 10:**
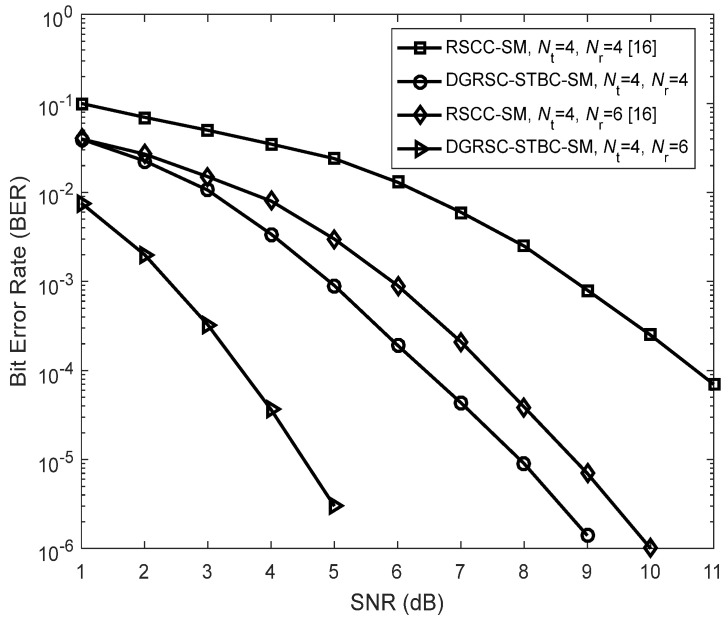
Performance comparisons between the DGRSC−STBC−SM scheme (using 4−QAM and **Algorithm 2**) and the existing RSCC−SM [16] scheme (using 16−QAM and random selection) under the conditions of $\lambda_{S,\;R}=\infty$, $C_{S}\;(63,\;51,\;13)$ and $C_{R}\;(63,\;31,\;33)$.

Firstly, the authors discuss the performance comparisons between the DGRSC-STBC-SM ($\lambda_{S,\;R}=\infty$) for our considered third case and the existing Reed–Solomon coded cooperative SM (RSCC-SM) [16] under $\lambda_{S,\;R}=\infty$. From Figure 10, we notice that under identical conditions such as the same spectral efficiency k/2=3 bits/s/Hz (k is shown in Equation (4)) and receive antenna number $N_{r}$, the DGRSC-STBC-SM provides better performance than the existing RSCC-SM. The reason behind such an enticing gain can be explained by using the following two aspects: (a) The STBC-SM technique adopted in our proposed DGRSC-STBC-SM scheme combines the ideas of STBC and SM, which is capable of removing the ICI and exploiting the transmit diversity. However, the SM technique used in the existing RSCC-SM scheme can only eliminate the ICI and is unable to exploit the transmit diversity. (b) In our proposed DGRSC-STBC-SM scheme, an efficient information selection **Algorithm 2** is used to properly select 31 symbols from 51 symbols. However, in the paper [16], the relay randomly selects 31 symbols from 51 symbols. Thus, employing **Algorithm 2** is very helpful for our proposed DGRSC-STBC-SM scheme to construct the codeword set with a larger minimum codeword weight (i.e., 50) due to the proper symbol selection at the relay. Observe from Figure 10 that at BER $\;\approx {1\times 10}^{-\;4}$, the DGRSC-STBC-SM using $N_{r}=4$ and 6 achieves SNR gains of about 4.3 dB and 3.9 dB, respectively, compared to the RSCC-SM scheme with $N_{r}=4$ and 6.

**Figure 11:**
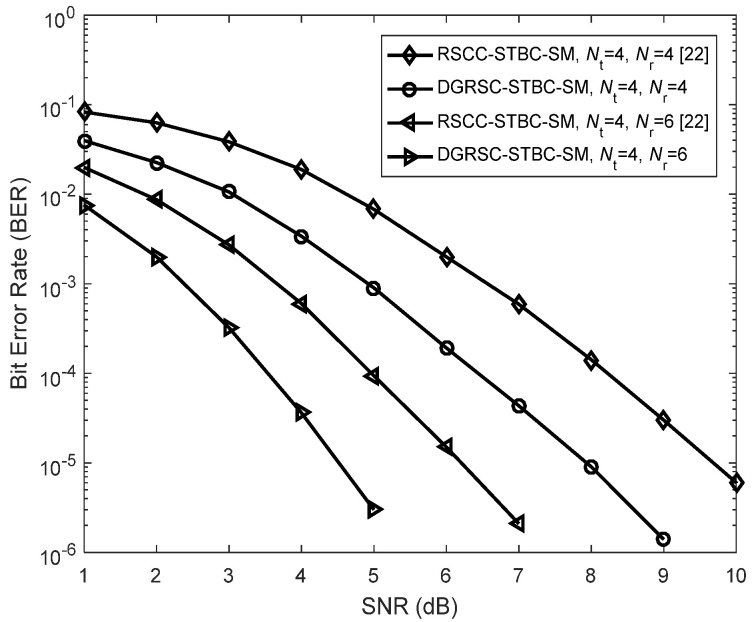
Performance comparisons between the DGRSC−STBC−SM scheme (using **Algorithm 2**) and the existing RSCC−STBC−SM [22] scheme (using random selection) under the conditions of 4−QAM, $\lambda_{S,\;R}=\infty$, $C_{S}\;(63,\;51,\;13)$ and $C_{R}\;(63,\;31,\;33)$.

Additionally, we compare our proposed DGRSC-STBC-SM scheme with the recent state-of-the-art RS-coded cooperative STBC-SM (RSCC-STBC-SM) scheme [22] under identical conditions. In our proposed DRSC-STBC-SM scheme, the optimized **Algorithm 2** is adopted at the relay to select 31 symbols from 51 source information symbols for further encoding. However, in the existing RSCC-STBC-SM scheme, 31 symbols are randomly chosen from 51 source information symbols for further encoding. As depicted in Figure 11, the DGRSC-STBC-SM scheme outperforms the existing scheme over the entire SNR under the same receive antenna number $N_{r}$. The reason for the excellent performance is based on the fact that our proposed system uses an optimized symbol selection algorithm to make the destination node generate an optimized codeword set with a larger minimum codeword weight.

### 7.4. Comparisons of DGRSC-STBC-SM Scheme with Different Numbers of Receiving Antennas

**Figure 12:**
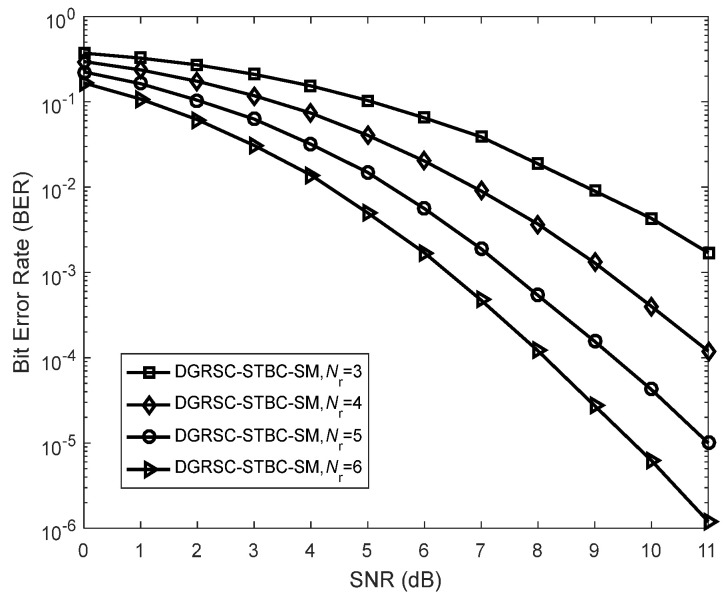
Error performance for DGRSC−STBC−SM scheme utilizing $C_{S}\;(10,\;5,\;6)$ and $C_{R}\;(10,\;3,\;8)$ with different numbers of receive antennas under **Algorithm 1**, $N_{t}=4$ and BPSK.

To see the effect of receive antenna number on the error performance of the DGRSC-STBC-SM scheme, this subsection demonstrates the performance comparisons of the DGRSC-STBC-SM scheme ($\lambda_{S,\;R}=\infty$) for the first case under different numbers of receiving antennas. From Figure 12, we observe that the effect of receive antenna number on the system error performance is very evident. Figure 12 also shows that the DGRSC-STBC-SM scheme under $N_{r}=6$ performs the best BER performance, but the BER performance of the DGRSC-STBC-SM scheme is the worst under $N_{r}=3$. The error performance under $N_{r}=4$ and 5 lies between the previous two. For example, at an SNR of 11 dB, the error performance under $N_{r}=6$ is $1{.2\times 10}^{-\;6}$. At the same SNR, the error performance under $N_{r}=4$ and 5 is $1{.2\times 10}^{-\;4}$ and ${1\times 10}^{-\;5}$, respectively. However, under $N_{r}=3$, poor error performance (i.e., $1{.7\times 10}^{-\;3}$) is generated at SNR=11 dB. Thus, this validates the fact that as the number of receiving antennas adds the overall system error performance is improved. The BER performance enhancement is because increasing the value of $N_{r}$ offers more spatial diversity to the DGRSC-STBC-SM scheme.

## 8. Conclusions

In this article, we propose the novel DGRSC-STBC-SM scheme with the information selection at the relay. For each selection at the relay, the destination generates a codeword set. To obtain the optimal codeword set (resulted by the best selection pattern) at the destination, we propose the optimal symbol selection algorithm at the relay. We also propose another optimized symbol selection algorithm with the low complexity to reduce the complexity of the optimal symbol selection algorithm. The simulation results demonstrate that the DGRSC-STBC-SM scheme using the proposed two optimized algorithms has better BER performance than that using the random selection pattern, which is because the codeword set with a larger minimum distance is generated at the destination by using the optimized algorithms. Furthermore, the DGRSC-STBC-SM scheme with the low-complexity symbol selection algorithm can obtain the near-optimal performance. Additionally, the numerical results confirm the superiority of the DGRSC-STBC-SM scheme over its corresponding non-cooperative counterpart. It is mainly because the relay-to-destination link has a larger SNR gain than the source-to-destination link. Moreover, our proposed DGRSC-STBC-SM scheme can significantly outperform the existing schemes by the use of the proper selection at the relay. Finally, we compare the BER performance of the DGRSC-STBC-SM scheme under the varying receive antenna number. The simulation results show that the system performance will be improved as the receive antenna number adds.

## Figures and Tables

**Table 1:** Mapping procedure for STBC-SM with codes over the field $F_{16}$.

Field Elements	Binary Vectors	*N*_t_ = 4, BPSK	
		Active TACs	Modulated Symbols
0	[0, 0, 0, 0]	(1, 2)	(−1, −1)
1	[1, 0, 0, 0]	(2, 3)	(−1, −1)
A	[0, 1, 0, 0]	(3, 4)	(−1, −1)
$\alpha^{2}$	[0, 0, 1, 0]	(1, 2)	(+1, −1)
$\alpha^{3}$	[0, 0, 0, 1]	(1, 2)	(−1, +1)
$\alpha^{4}$	[1, 1, 0, 0]	(4, 1)	(−1, −1)
$\alpha^{5}$	[0, 1, 1, 0]	(3, 4)	(+1, −1)
$\alpha^{6}$	[0, 0, 1, 1]	(1, 2)	(+1, +1)
$\alpha^{7}$	[1, 1, 0, 1]	(4, 1)	(−1, +1)
$\alpha^{8}$	[1, 0, 1, 0]	(2, 3)	(+1, −1)
$\alpha^{9}$	[0, 1, 0, 1]	(3, 4)	(−1, +1)
$\alpha^{10}$	[1, 1, 1, 0]	(4, 1)	(+1, −1)
$\alpha^{11}$	[0, 1, 1, 1]	(3, 4)	(+. 1, +1)
$\alpha^{12}$	[1, 1, 1, 1]	(4, 1)	(+1, +1)
$\alpha^{13}$	[1, 0, 1, 1]	(2, 3)	(+1, +1)
$\alpha^{14}$	[1, 0, 0, 1]	(2, 3)	(−1, +1)

**Table 2:** Codeword weight ${\mathrm{wt}(|c|c}_{j}\left|\right)$ at the destination in ascending order.

${\mathrm{wt}(|c|c}_{j}\left|\right)$	$\left({\mathrm{wt}(c),\;\mathrm{wt}(c}_{j})\right)$
3	(3, 0)
4	(4, 0)
5	(5, 0)
7	(3, 4)
8	(3, 5), (4, 4)
9	(4, 5), (5, 4)
10	(5, 5)

**Table 3:** Number of codewords $B_{w}^{\left(\;j\;\right)}$ with ${\mathrm{wt}(|c|c}_{j}|)=w$ for $\Omega_{j}$.

$\Omega_{j}$	$B_{3}^{\;(\;j\;)}$	$B_{4}^{\;(\;j\;)}$	$B_{5}^{\;(\;j\;)}$	$B_{7}^{\;(\;j\;)}$
$\Omega_{1}$	0	0	7	56
$\Omega_{2}$	0	0	7	49
$\Omega_{3}$	0	0	7	56

**Table 4:** Process of choosing J elements.

wt(*c*) = *i*	*J*	1st Part: 10 − *i*	2nd Part: *J* − (10 − *i*)
6	4	4	0
7	3	3	0
	4	3	1
8	2	2	0
	3	2	1
	4	2	2
9	1	1	0
	2	1	1
	3	1	2
	4	1	3
10	0	0	0
	1	0	1
	2	0	2
	3	0	3
	4	0	4

**Table 5:** Codeword weight ${\mathrm{wt}(|c|c}_{j}\left|\right)$ at the destination in an ascending order.

wt(|*c*|*c_j_*|)	(wt(*c*), wt(*c_j_*))
6	(6, 0)
7	(7, 0)
8	(8, 0)
9	(9, 0)
10	(10,0)
14	(6, 8)
15	(6,9), (7,8)
16	(6,10), (7,9), (8,8)
17	(6,11), (7,10), (8,9), (9,8)
18	(6,12), (7,11), (8,10), (9,9),(10,8)
19	(6,13), (7,12), (8,11), (9,10), (10,9), (11,8)
20	(6,14), (7,13), (8,12), (9,11), (10,10), (11,9), (12,8)

**Table 6:** Relationship between the number ${\bar{B}}_{w}^{\left(j\right)}$ of codeword weight ${\mathrm{wt}(|c|c}_{j}|)=w$ and $\Omega_{j}$.

$\Omega_{j}$	${\bar{B}}_{6}^{\left(j\right)}$	${\bar{B}}_{7}^{\left(j\right)}$	${\bar{B}}_{8}^{\left(j\right)}$	${\bar{B}}_{9}^{\left(j\right)}$
$\Omega_{1}$	0	0	0	90
$\Omega_{2}$	0	0	15	—
$\Omega_{3}$	0	0	0	90
$\Omega_{4}$	0	0	0	90
$\Omega_{5}$	0	0	0	60
$\Omega_{6}$	0	0	0	9
$\Omega_{7}$	0	0	0	10

**Table 7:** Complexity comparison between Algorithm 1 and Algorithm 2.

Parameters	Algorithm 1	Algorithm 2	Percentage Reduction, %
$C_{S}\left(5,\;3,\;3\right)$ $,\;C_{R}\left(5,\;2,\;4\right)$ $,\;q=8,\;{\;k}_{b}=320$ $N_{\;R,\;3}^{\;(\mathrm{opt})}=N_{\;R,\;4}^{\;(\mathrm{opt})}=N_{\;R,\;5}^{\;(\mathrm{opt})}=N_{\;R,\;7}^{\;(\mathrm{opt})}=3$ ${\;N}_{\;R,\;3}^{\;(\mathrm{low})}=N_{\;R,\;4}^{\;(\mathrm{low})}=N_{\;R,\;5}^{\;(\mathrm{low})}=N_{\;R,\;7}^{\;(\mathrm{low})}=2$	104,960	46,400	56
$C_{S}\left(10,\;5,\;6\right)$ $,\;C_{R}\left(10,\;3,\;8\right)$ $,\;q=16,\;{\;k}_{b}=489001$ $N_{\;R,\;6}^{\;(\mathrm{opt})}=N_{\;R,\;7}^{\;(\mathrm{opt})}=N_{\;R,\;8}^{\;(\mathrm{opt})}=10$ $N_{\;R,\;6}^{\;(\mathrm{low})}=N_{\;R,\;7}^{\;(\mathrm{low})}=N_{\;R,8}^{\;(\mathrm{low})}=7$ $,\;N_{\;R,\;9}^{\;(\mathrm{low})}=6$	1,667,235,840	704,161,440	58

**Table 8:** Parameter vectors corresponding to the three cases.

Cases	$C_{S}{(n}_{1}{,\;k}_{1}{,\;d}_{1}),\;$ $C_{R}{(n}_{2}{,\;k}_{2}{,\;d}_{2})$	Parameter Vectors
1	$C_{S}\left(10,\;5,\;6\right),\;$ $C_{R}\left(10,\;3,\;8\right)$	$\alpha_{S}{=[\alpha ,\;\alpha }^{2}{,\;\alpha }^{3}{,\;\alpha }^{4}{,\;\alpha }^{5}{,\;\alpha }^{6}{,\;\alpha }^{8}{,\;\alpha }^{9}{,\;\alpha }^{12}{,\;\alpha }^{7}]$ $,\;v_{S}{=[\alpha ,\;\alpha }^{2}{,\;\alpha }^{3}{,\;\alpha }^{4}{,\;\alpha }^{4}{,\;\alpha }^{5}{,\;\alpha }^{8}{,\;\alpha }^{7}{,\;\alpha }^{10}{,\;\alpha }^{10}]$ $\alpha_{R}{=[1,\;\alpha ,\;\alpha }^{2}{,\;\alpha }^{4}{,\;\alpha }^{6}{,\;\alpha }^{5},$ $\alpha^{7}{,\;\alpha }^{9}{,\;\alpha }^{10}{,\;\alpha }^{11}]$ $,\;v_{R}{=[1,\;\alpha ,\;\alpha }^{2}{,\;\alpha }^{3}{,\;\alpha }^{5}{,\;\alpha }^{5}{,\;\alpha }^{7}{,\;\alpha }^{5}{,\;\alpha }^{8}{,\;\alpha }^{9}]$
2	$C_{S}\left(25,\;19,\;7\right),\;$ $C_{R}\left(25,\;10,\;16\right)$	$\alpha_{S}=[\gamma ,\;\gamma^{2},\;\gamma^{3},\;\gamma^{4},\;\gamma^{5},\;\gamma^{6},\;\gamma^{8},\;\gamma^{9},\;\gamma^{12},\;\gamma^{7},\;\gamma^{10},\;\gamma^{11},\;\gamma^{13},\;\gamma^{14},\;\gamma^{15},\;\gamma^{18},\;\gamma^{19},\;\gamma^{20},\;\gamma^{21},\;\gamma^{25},\;\gamma^{26},\;\gamma^{27},\;\gamma^{29},\;\gamma^{30},\;1]$
		$v_{S}=[\gamma ,\;\gamma^{3},\;\gamma^{3},\;\gamma^{4},\;\gamma^{5},\;\gamma^{6},\;\gamma^{7},\;\gamma^{8},\;\gamma^{9},\;\gamma^{12},\;\gamma^{7},\;\gamma^{10},\;\gamma^{11},\;\gamma^{12},\;\gamma^{13},\;\gamma^{15},\;\gamma^{15},\;\gamma^{17},\;\gamma^{19},\;\gamma^{20},\;\gamma^{21},\;\gamma^{24},\;\gamma^{26},\;\gamma^{27},\;\gamma^{29}]$
		$\alpha_{R}=[1,\;\gamma ,\;\gamma^{2},\;\gamma^{3},\;\gamma^{4},\;\gamma^{5},\;\gamma^{6},\;\gamma^{8},\;\gamma^{9},\;\gamma^{12},\;\gamma^{7}$ $,\;\gamma^{10},\;\gamma^{11},\;\gamma^{13},\;\gamma^{14},\;\gamma^{17},\;\gamma^{18},\;\gamma^{19},\;\gamma^{20},\;\gamma^{21},\;\gamma^{25},\;\gamma^{26},\;\gamma^{27},\;\gamma^{29},\;\gamma^{30}]$
		$v_{R}=[1,\;\gamma ,\;1,\;\gamma^{3},\;\gamma^{4},\;\gamma^{5},\;\gamma^{6},\;\gamma^{8},\;\gamma^{9},\;\gamma^{11},\;\gamma^{7},\;\gamma^{10},\;\gamma^{11},\;\gamma^{13},\;\gamma^{14},\;\gamma^{17},\;\gamma^{17},\;\gamma^{19},\;\gamma^{21},\;\gamma^{21},\;\gamma^{25},\;\gamma^{26},\;\gamma^{27},\;\gamma^{29},\;\gamma^{30}]$
3	$C_{S}\left(63,\;51,\;13\right),\;$ $C_{R}\left(63,\;31,\;33\right)$	$\alpha_{S}{=v}_{S}{=\alpha }_{R}{=v}_{R}=[1,\;\chi ,\;\ldots ,\;\chi^{62}]$

**Table 9:** Parameters utilized in the simulation.

Parameters	Specification
Source coding	$C_{S}\;(10,\;5,\;6)$ $,\;C_{S}\;(25,\;19,\;7)$ $,\;C_{S}\;(63,\;51,\;13)$
Relay coding	$C_{R}\;(10,\;3,\;8)$ $,\;C_{R}\;(25,\;10,\;16)$ $,\;C_{R}\;(63,\;31,\;33)$
Effective code rate of destination	1/4, 19/50, 51/126
Channel model	Slow Rayleigh fading channel
MIMO configuration	STBC-SM: $N_{t}=4$$,\;\mathrm{BPSK},\;N_{r}=3$, 4, 5, 6 $N_{t}=3$, 4-QAM, $N_{r}=4$ $N_{t}=4$, 4-QAM, $N_{r}=4$, 6 $\mathrm{SM}:\;N_{t}=4$, 16-QAM, $N_{r}=4$, 6
MIMO detection	Maximum likelihood (ML) detection
GRS decoding algorithm	Euclidean decoding algorithm

**Table 10:** Optimized selection patterns corresponding to three different cases.

Cases	$\eta^{\left(\mathrm{opt}\right)}$	$\eta^{\left(\mathrm{low}\right)}$
1	[2, 3, 4]	[1, 3, 4]
2	——	[5, 8, 9, 10, 12, 13, 14, 15, 17, 18]
3	——	[0, 1, 2, 3, 4, 5, 6, 11, 12, 13, 16, 18, 19, 22, 24, 25, 26, 27, 29, 31, 33, 34, 35, 40, 42, 44, 45, 47, 48, 49, 50]

## Data Availability

Not applicable.

## References

[1] Guo S., Zhang H., Zhang P., Dang S., Liang C., Alouini M.S. (2019). Signal Shaping for Generalized Spatial Modulation and Generalized Quadrature Spatial Modulation. IEEE Trans. Wirel. Commun..

[2] Wu Y., Ying H., Jiang X., Hai H. (2019). A Joint Data Mapping and Detection for High Performance Generalized Spatial Modulation. IEEE Commun. Lett..

[3] Huang K., Xiao Y., Liu L., Li Y., Song Z., Wang B., Li X. (2022). Integrated Spatial Modulation and STBC-VBLAST Design Toward Efficient MIMO Transmission. Sensors.

[4] Feng D., Xu H., Zheng J., Bai B. (2018). Nonbinary LDPC-Coded Spatial Modulation. IEEE Trans. Wirel. Commun..

[5] Basar E., Aygolu U., Panayirci E., Poor H.V. (2011). Space-Time Block Coded Spatial Modulation. IEEE Trans. Commun..

[6] Ejaz S., Yang F., Xu H. (2020). Split Labeling Diversity for Wireless Half-Duplex Relay Assisted Cooperative Communication Systems. Telecommun. Syst..

[7] Zhao C., Yang F., Umar R., Mughal S. (2020). Two-Source Asymmetric Turbo-Coded Cooperative Spatial Modulation Scheme with Code Matched Interleaver. Electronics.

[8] Mesleh R., Ikki S.S. (2019). Performance Analysis of Spatial Modulation with Multiple Decode and Forward Relays. IEEE Wirel. Commun. Lett..

[9] Hai H., Li C., Peng Y., Hou J., Jiang X. (2021). Space-Time Block Coded Cooperative MIMO Systems. Sensors.

[10] Hu J., Duman T.M. (2007). Low Density Parity Check Codes over Wireless Relay Channels. IEEE Trans. Wirel. Commun..

[11] Qiu J., Liu S. (2020). A Novel Concatenated Coding Scheme: RS-SC-LDPC Codes. IEEE Commun. Lett..

[12] Niu Y., Yue Q., Wu Y., Hu L. (2019). Hermitian Self-Dual, MDS, and Generalized Reed-Solomon Codes. IEEE Commun. Lett..

[13] Sun R., Tian Y., Liu J. Construction of QC-LDPC Codes Based on Generalized RS Codes with Girth Larger Than 6. Proceedings of the International Conference on Communication Systems.

[14] Jin L., Xing C. (2017). New MDS Self-Dual Codes from Generalized Reed-Solomon Codes. IEEE Trans. Inf. Theory.

[15] Chen B., Liu H. (2018). New Constructions of MDS Codes with Complementary Duals. IEEE Trans. Inf. Theory.

[16] Zhao C., Yang F., Waweru D.K. (2021). Reed-Solomon Coded Cooperative Spatial Modulation Based on Nested Construction for Wireless Communication. Radioengineering.

[17] Mughal S., Yang F., Xu H., Umar R. (2018). Coded Cooperative Spatial Modulation Based on Multi-Level Construction of Polar Code. Telecommun. Syst..

[18] Mughal S., Yang F., Xu H., Umar R. (2017). Polar Coded Space-Time Block Coded Spatial Modulation Based on Plotkin’s Construction. IET Commun..

[19] Almawgani A.H.M., Salleh M.F.M. RS Coded Cooperation with Adaptive Cooperation Level Scheme over Multipath Rayleigh Fading Channel. Proceedings of the IEEE 9th Malaysia International Conference on Communications (MICC).

[20] Almawgani A.H.M., Salleh M.F.M. (2010). Coded Cooperation Using Reed Solomon Codes in Slow Fading Channel. IEICE Electron. Expr..

[21] Al-moliki Y.M., Aldhaeebi M.A., Almwald G.A., Shaobi M.A. The Performance of RS and RSCC Coded Cooperation Systems Using Higher Order Modulation Schemes. Proceedings of the 6th International Conference on Intelligent Systems, Modelling and Simulation.

[22] Zhao C., Yang F., Chen C., Umar R. Reed-Solomon Coded Cooperative Space-Time Block Coded Spatial Modulation. Proceedings of the International Conference on Wireless Communications and Smart Grid (ICWCSG) 2021.

[23] MacWilliams F.J., Sloane N.J.A. (1977). The Theory of Error-Correcting Codes.

[24] Guo P., Yang F., Zhao C., Ullah W. (2021). Jointly Optimized Design of Distributed Reed–Solomon Codes by Proper Selection in Relay. Telecommun. Syst..

[25] Varshney N., Krishna A.V., Jagannatham A.K. (2015). Selective DF Protocol for MIMO STBC Based Single/Multiple Relay Cooperative Communication: End-to-End Performance and Optimal Power Allocation. IEEE Trans. Commun..

